# Supplementary material for: Does multidisciplinary videoconferencing between a head-and-neck cancer centre and its partner hospital add value to their patient care and decision-making? A mixed-method evaluation
Source: BMJ Open. 2019 Nov 7;9(11):e028609. doi: 10.1136/bmjopen-2018-028609 (PMC6858233; doi:10.1136/bmjopen-2018-028609)
Supplement: Author's manuscript [file bmjopen-2018-028609.draft_revisions.pdf]

BMJ Open is committed to open peer review. As part of this commitment we make the peer review history of every article we publish publicly available.

When an article is published we post the peer reviewers' comments and the authors' responses online. We also post the versions of the paper that were used during peer review. These are the versions that the peer review comments apply to.

The versions of the paper that follow are the versions that were submitted during the peer review process. They are not the versions of record or the final published versions. They should not be cited or distributed as the published version of this manuscript.

BMJ Open is an open access journal and the full, final, typeset and author-corrected version of record of the manuscript is available on our site with no access controls, subscription charges or pay-per-view fees (http://bmjopen.bmj.com).

If you have any questions on BMJ Open's open peer review process please email info.bmjopen@bmj.com

BMJ Open

Does multidisciplinary videoconferencing between a head and neck cancer centre and its partner hospital add value to their patient care and decision making? A mixed method evaluation

Journal:	*BMJ Open*
Manuscript ID	bmjopen-2018-028609
Article Type:	Research
Date Submitted by the Author:	19-Dec-2018

Complete List of Authors:	van Huizen, Lidia; University of Groningen, University Medical Center Groningen, Quality and Patient Safety; Oral and Maxillofacial Surgery<br>Dijkstra, Pieter; University of Groningen, University Medical Center Groningen, Centre for Rehabilitation<br>Halmos, Gyorgy; University of Groningen, University Medical Center Groningen, Ear, Nose and Throat<br>van den Hoek, Johanna; University of Groningen, University Medical Center Groningen, Radiotherapy<br>van der Laan, Klaas; Medical Center Leeuwarden, Ear, Nose and Throat<br>Wijers, Oda; Radiotherapeutic Institute Friesland<br>Ahaus, Kees; University of Groningen, Faculty of Economics and Business, Operations; University of Groningen, Faculty of Economics and Business,<br>de Visscher, Jan; Medical Center Leeuwarden, Oral and Maxillofacial Surgery; Free University Medical Center, Oral and Maxillofacial Surgery / Oral Pathology<br>Roodenburg, Jan; University of Groningen, University Medical Center Groningen, Oral and Maxillofacial Surgery

Keywords:	Videoconferencing, Collaborating teams, Mixed method study, Added value, Multidisciplinary treatment, Head & neck tumours < ONCOLOGY

SCHOLARONE™ Manuscripts

Does multidisciplinary videoconferencing between a head and neck cancer centre and its partner hospital add value to their patient care and decision making? A mixed method evaluation

Lidia S. van Huizen (LvH), MSc[1,2], corresponding author, l.s.van.huizen@umcg.nl, +31 50 361 8035

Pieter U. Dijkstra (PD), PT, PhD[1,3], p.u.dijkstra@umcg.nl

Gyorgy B. Halmos (GH), MD, PhD[4], g.b.halmos@umcg.nl

Johanna G.M. van den Hoek (JvdH), MD[5], j.g.m.van.den.hoek@umcg.nl

Klaas T. van der Laan (KvdL), MD, PhD[6], kens.vdlaan@planet.nl

Oda B. Wijers (OW), MD, PhD[7], o.b.wijers@skf-rif.nl

Kees (C.)T. B. Ahaus (KA), PhD[8], c.t.b.ahaus@rug.nl

Jan G.A.M. de Visscher (JdV), DMD, MD, PhD[9, 10], j.de.visscher@znb.nl

Jan L.N. Roodenburg (JR), DDS, MD, PhD[1], j.l.n.roodenburg@umcg.nl

[1] University of Groningen, University Medical Center Groningen, Department of Oral and Maxillofacial Surgery, Groningen, The Netherlands

[2] University of Groningen, University Medical Center Groningen, Department of Quality and Patient Safety, Groningen, The Netherlands

[3] University of Groningen, University Medical Center Groningen, Centre for Rehabilitation, Groningen, The Netherlands

[4] University of Groningen, University Medical Center Groningen, Department of Ear, Nose & Throat / Head and Neck Surgery, Groningen, The Netherlands

[5] University of Groningen, University Medical Center Groningen, Department of Radiotherapy, Groningen, The Netherlands

[6] Medical Centre Leeuwarden, Department of Ear, Nose & Throat, Leeuwarden, The Netherlands

[7] Radiotherapeutic Institute Friesland, Leeuwarden, the Netherlands

[8] University of Groningen, Faculty of Economics and Business, Centre of Expertise Healthwise, University Medical Center Groningen, Groningen, The Netherlands

For peer review only - http://bmjopen.bmj.com/site/about/guidelines.xhtml

[9] Medical Centre Leeuwarden, Department of Oral and Maxillofacial Surgery, Leeuwarden, The Netherlands

[10] Free University Medical Center, Department of Oral and Maxillofacial Surgery / Oral Pathology, Amsterdam, The Netherlands

ABSTRACT (wordcount 300)

Objectives

Given the difficulties in diagnosing and treating head and neck cancer, care is centralized in the Netherlands in eight head and neck cancer centres and six satellite regional hospitals as preferred partners. A requirement is that all patients cases of the partners should be discussed with the head and neck centre as part of the 'collaborating contract'. In this mixed method study, we evaluate the added value of videoconferencing regarding recommendations given and benefits for patients and teams.

Design

For one centre and its partner recommendations exchanged between their multidisciplinary team were registered over six months. Semi-structured interviews were held with six medical specialists, three from the centre and three from the partner, to explore the perceived benefits and drawbacks of multidisciplinary videoconferencing.

Results

In total 336 patient cases were presented. In 8 cases (2%), specialists offered recommendations to the collaborating team (3 given from centre to partner and 5 from partner to centre), that mainly consisted of alternative diagnostic modalities or treatment plans for a specific patient. The interviews on the videoconferencing revealed that medical specialists perceive added value in discussing complex cases because the other team offers a fresh perspective by hearing the case 'as new'. The teams recognize the importance of keeping their medical viewpoints aligned, but the requirement (the partner should discuss all patients with the centre) was seen as out-dated and lacking added value.

Conclusions

The added value of the videoconferencing is small considering patient care, but the specialists recognize that it is important that their medical viewpoints are aligned and that their patients benefit

For peer review only - http://bmjopen.bmj.com/site/about/guidelines.xhtml

from the discussions about complex cases. Both, centre and partner felt the videoconferencing meeting could be more efficiently organized.

We suggest to teams who want to implement videoconferencing as supportive medium: meet face-to-face and make agreements before you start.

Keywords

Videoconferencing (MeSH term), head and neck cancer, collaborating teams, multidisciplinary treatment, added value, mixed method study

Strengths and limitations of this study

- Videoconferencing facilitates multidisciplinary meetings between collaborating teams from different locations to discuss complex cases that will benefit the quality of patient care and the treating teams in keeping their medical viewpoints aligned (strength)
- The videoconferencing process could be more efficient if clear-cut cases that fully fit the national multidisciplinary guidelines on diagnostic and treatment plans did not have to be discussed between the centre and the partner (strength)
- Participating specialists from different specialisms and locations were interviewed and identified benefits and drawbacks of the videoconference meetings (strength)
- Only one of the six centres and its preferred partner in the Netherlands was studied in depth (limitation)
- The researcher's presence may have influenced the 'recommendation culture' (limitation)

Wordcount 3997, without tables

INTRODUCTION

Most tumours in the head or neck region (nasal cavity, paranasal sinuses, lips, mouth, salivary glands, throat or larynx and complex skin malignancies) are fast growing tumours. This implies that a long interval between the moment of referral and the start of the primary treatment (surgery, radiotherapy and/or chemotherapy) can lead to tumour progression with less survival chance[1]. Because of the complexity of the diagnostic procedures and therapeutic modalities and the low volume of patients, head and neck cancer care is centralized in multidisciplinary head and neck cancer centres (high complex low volume cases)[2]. In 1984, the Dutch Head and Neck Society (DHNS) was founded as a

For peer review only - http://bmjopen.bmj.com/site/about/guidelines.xhtml

For peer review only

scientific organization. Later the DHNS became involved in the nationwide organization of head and neck cancer care. As a result, since 1993, head and neck cancer patients in the Netherlands are treated in eight head and neck cancer centres recognized by the DHNS, of which six have preferred partners[3]. Within each head and neck cancer centre, multidisciplinary meetings according to national evidence-based guidelines are mandatory to provide the best diagnostic work up and treatment for patients, and to sustain the quality of care in the oncology centres[4-7].

In 1997, after a pilot of 4 years , the Medical Centre Leeuwarden became the preferred partner of the Head and Neck Cancer Centre of the University Medical Centre Groningen[8], further referred to as the "partner" and the "centre". The collaboration of a centre with its partner is based upon trust and sustainable agreements[9-11]. The backbone of the collaboration is the weekly multidisciplinary meeting between centre and partner to discuss and verify diagnostic and therapeutic plans. The efficiency of the multidisciplinary meetings is important for decision making and care pathway management[12].

The DHNS and the Dutch Health Care Inspectorate (DHCI) require that all new patients of the partner, including those with relatively common head and neck tumours, be discussed in a weekly multidisciplinary meeting with the centre[2]. This requirement can be seen as a form of quality control over the partner clinic (further referred to as 'the DHCI requirement'). Specialists from both locations, centre and partner, from the departments of oral and maxillofacial surgery (OMS), ear, nose and throat (ENT) and radiotherapy (RT) participate. This weekly multidisciplinary meeting is additional to a local multidisciplinary patient meeting held at the hospital where the patient is first seen and will be treated. Initially, these collaborative multidisciplinary weekly meetings were held in the centre but, when videoconferencing became available, this medium became the method of choice for this communication[13, 14]. The weekly videoconference is scheduled after the local multidisciplinary meetings. During the videoconferencing, the partner presents all patient cases, including available imaging, and proposed diagnostic and therapeutic plan. The centre presents a selection of its cases, those it considers complex or interesting to discuss. Both sides are free to offer recommendations. The team that presents the patient case is responsible for documenting changes when a recommendation is implemented. Recommendations from both teams to the decision-making regarding diagnostic and therapeutic plans may add value to the quality of patient care[15].

For peer review only - http://bmjopen.bmj.com/site/about/guidelines.xhtml

Research Question

Our overall question was whether the multidisciplinary videoconferencing between a head and neck centre and its partner adds value to the treatment of head and neck cancer patients in the care pathways. This resulted in two sub questions.

1. In what percentage of cases recommendations are given on diagnostic and/or therapeutic plans being given by the teams during the videoconference?
2. What benefits and drawbacks of the videoconference are perceived by the specialists in the teams?

DESIGN

In this mixed method study, we evaluate the added value of this multidisciplinary videoconferencing for head and neck cancer care or pathway management. The mixed method approach[16-18] had quantitative and qualitative elements. The primary outcome of the weekly multidisciplinary videoconferencing, 'added value', was first operationalized as the percentage of cases in which recommendations on diagnostic and treatment plans were given. A second component of 'added value' was operationalized as the benefits or drawbacks experienced by the participants of the multidisciplinary videoconferencing. In the study period, the teams acted conform the DHCI requirement that all patients of the partner should be presented in a multidisciplinary meeting with the centre.

Patient data

Data of all patients presented by one of the teams during the weekly multidisciplinary videoconferences between September 2016 and February 2017 were included. The tumour localization, histology and tumour stage were registered for all patients that were presented.

Recommendation registration

Recommendations made during the videoconference were registered with the relevant data from electronic and written medical records on a clinical registration form by LvH during the videoconference. Each recommendation was assessed by the two teams with respect to change impact (minor or major, Table 1a) on the diagnostic and/or therapeutic plan, case complexity, use of national multidisciplinary guidelines for diagnostic or treatment plans, and comorbidity of the patient (Table

For peer review only - http://bmjopen.bmj.com/site/about/guidelines.xhtml

1b)[19, 20]. LvH registered the given recommendation with the relevant data; JdV and JR verified the registrations. During the videoconferencing sessions, field notes were taken by LvH.

Table 1. Definitions of change impact and case complexity

Table 1a Operational definitions of major and minor changes in diagnostic or treatment plan

	diagnostic plan	treatment plan	remarks
minor	additional more-detailed MRI or CT-Thorax of the area already imaged	logistic change	
major	additional MRI or CT-thorax in a different area from the area already imaged	change in modality: adding or deleting a therapeutic modality; surgery radiotherapy or chemotherapy	
criterion	addition of diagnostic plan in a different area than already investigated	adding or deleting a treatment modality from the treatment plan in the proposed or in a different area	after the major/minor decision is made, the decision registered in the CRF will be verified by both specialists (giver and receiver).

Table 1b Operational definition of case complexity

	modality	guideline	comorbidity
not complex	unimodal treatment	diagnosis and treatment follows guideline	no comorbidity
complex	multimodal treatment	diagnosis and/or treatment does not follow guideline	comorbidity
remark	- unimodal:<br>surgical procedure<br>chemotherapy<br>primary radiotherapy<br>- multimodal:<br>reconstruction surgery<br>chemo- or bioRT	which guidelines are followed	

Patient involvement in study design

Patients were not involved in the study design because the main purpose of the study was to evaluate the added value of the DHCI requirement to discuss all patients of the partner with the centre in a weekly videoconference.

Qualitative Analysis

Semi-structured interviews were conducted with six medical specialists, one from the OMS-, ENT- and RT-department of the centre and the partner, to explore the added value of the weekly

For peer review only - http://bmjopen.bmj.com/site/about/guidelines.xhtml

For peer review only

videoconference. The field notes taken by the researcher during the weekly videoconference sessions were used to develop the questions for the semi-structured interviews. After receiving verbal informed consent from the specialists, the semi-structured interviews started with providing information about the recommendations given. Thereafter it continued with the open question ‘What do you think is the value of the videoconference between the head and neck cancer centre and their preferred partner?’. LvH then guided the interview using a short topic list including ‘added value’ and ‘perceived possibilities for change or improvement in the videoconferencing process’ (Textbox 1). The different topics were introduced in a flexible way, and the interviews took the form of natural conversations.

Textbox 1 Interview Guide

Topics	Questions
Added value videoconferencing	What do you think is the added value of the videoconference between the head and neck cancer centre (centre) and their preferred partner (partner)?<br>Could you mention strong points of the videoconferencing?<br>Could you give examples?<br>Could you name points for improvement?<br>Could you mention examples?
Role of specialism in videoconference	What do you think the role of a specialist is in the videoconferencing between centre and partner?<br>The consultation is required by the Dutch Head and Neck Working Group and the Dutch Health Inspectorate, how usefulness do you think it is?<br>Would you advise stopping the consultation if it was not mandatory?
Results interpretation	Have you given recommendations to the centre/partner?<br>Have you received recommendations from the centre/partner?<br>Could you indicate what the difference is between peer consultation and giving a recommendation?<br>What do you think would be an ideal videoconference? Could you explain your answer?<br>What do you think could be adjusted in the videoconference to make the consultation more effective and more efficient?

Interviews took 25 to 40 minutes, were audio recorded and transcripts of the interviews were made. The participants were asked to review the transcript report and extracted quotes. These quotes related to perceived added value, possible improvements and the role of a specialist in the multidisciplinary videoconference. Following this, quotes were coded for their relevance to possible benefits or drawbacks for the collaboration between the teams and for patient care. The first stage of this inductive analysis of the interviews involved two authors, JR and JdV, in an initial open coding procedure that resulted in a list of codes corresponding closely to the text fragments extracted from the six interviews. The codes were placed in a coding tree in relation to the research question[21]. Any

For peer review only - http://bmjopen.bmj.com/site/about/guidelines.xhtml

disagreements during the coding were discussed between the coders and the researcher[22]. After the preliminary results were collated, a member check was performed to check credibility of results with participants [23].

Sample size calculation

In a pilot study for the quantitative part of the study, it was found that approximately 20% of the cases presented at the videoconference led to a recommendation. To estimate this percentage to a 10% precision (95 % confidence interval: 15.5 % to 25.4 %) would require a total of 250 cases. On average, 15 cases were discussed at each weekly videoconference meeting and, on this basis, we calculated that six months would be sufficient to acquire the necessary 250 cases.

The pilot study was also used to define and operationalize the different options for measurements to answer the quantitative research question.

Videoconferencing equipment used

Videoconferencing takes place in dedicated multidisciplinary meeting rooms, where two screens can be operated with two to four computers with monitors. The videoconferencing is operated via application 'Webex' and a camera, one of the locations calls the other. While the patient data is presented on the first screen, teams can see each other on the second screen. Data is sent via obtical fiber.

Statistical analysis

Our primary outcome measure was the percentage of cases presented that led to recommendations by the team of the centre to the partner or vice versa. The t-test for independent samples and the Chi-Square test (exact procedure case requirements for the Chi-Square test were not met) was used to analyse differences in age, gender, tumour localization and tumour histology (ICD(O))[24], and tumour stage between cases presented by the centre and those presented by the partner. Statistical analyses were performed using SPSS 23.0 for Windows software. In all analyses, statistical significance was set at the 5% level.

RESULTS

Quantitative analysis

For peer review only - http://bmjopen.bmj.com/site/about/guidelines.xhtml

From September 2016 to February 2017, 82 patients were presented by the centre and 177 by the partner in 18 weekly videoconferencing meetings (Table 2). In this period of 22 weeks, three meetings were cancelled due to a ‘medical complication meeting’, a technical problem to connect and a holiday recess; on one occasion the researcher could not attend the meeting.

For peer review only - http://bmjopen.bmj.com/site/about/guidelines.xhtml

Table 2 Patients and their tumour characteristics, as presented during videoconference meetings

Number of patients (total n = 259)	*centre (n = 82)*		*partner (n = 177)*		statistics, p =
(n= number of available data)	*mean*	*sd*	*mean*	*sd*	
**Age** (Mean, sd)	67.8	15.2	66.7	16.1	*(t-test) .533*
**Gender (n = 259)**	*n*	*%*	*n*	*%*	*(Chi^2) .394*
Female	27	10	68	26	
**Tumour localization (n = 206*)**	*n*	*%*	*n*	*%*	*(Chi^{2-}exact) < .001*
Lip (C00)	3	3	4	2	
Oral cavity	21	23	29	12	
Tongue (C01, C02)	6	-	11	-	
Gums (C03)	5	-	7	-	
Floor of mouth (C04)	4	-	4	-	
Oral cavity, unspecified (C05, C06, C14)	6	-	7	-	
Major salivary glands (C07, C08)	2	2	7	3	
Oropharynx (C09,C10)	7	8	6	2	
Nasopharynx (C11)	0	0	0	0	
Nasal Cavity (C30)	2	2	3	1	
Hypopharynx (C12, C13)	5	5	5	2	
Sinus (C31)	3	3	3	1	
Larynx (C32)	10	11	15	6	
Bronchus and Lung (C34)	0	0	5	2	
Hematologic and reticuloendothelial systems (C42)	0	0	11	5	
Skin (C44)	14	15	35	14	
Lymph nodes (C77)	2	2	1	0	
Unknown (C80)	3	3	0	0	
Miscellaneous (C20, 33, 41, 49, 50, 64, 73)	3	3	7	3	
Unknown (C80)	3	3	0	0	
**Morphology or cell type (n = 259)**	*n*	*%*	*n*	*%*	*(Chi^2) < .001*
Squamous cell carcinoma (SCC)	57	72	78	44	
Basic cell carcinoma (BCC)	3	4	6	3	
Melanoma	0	0	11	6	
Miscellaneous malignant	7	9	9	5	
Benign	2	2	18	10	
Infection – premalignant abnormalities	2	2	12	7	
Miscellaneous	11	13	43	24	
**T-stage (n= 159**)**	*n*	*%*	*n*	*%*	*(Chi^2) < .001*
T1	13	14	42	17	
T2	20	22	20	8	
T3	8	9	9	4	
T4	25	27	14	6	
Tx	7	8	1	1	

*= only tumour localization if tumour diagnosed; **= only TNM-code if firstly diagnosed, so there are more patients in which 'localization' is known (i.e. for relapse or tumour residue or metastases).93
In total 336 cases presented: 93 by centre and 243 by partner.

For peer review only

For peer review only - http://bmjopen.bmj.com/site/about/guidelines.xhtml

Most of the centre's patients (71 out of 82 – 86%) were presented only once, 9 were presented twice (11 %), one patient was discussed three times and another four times. Whereas 111 patients were presented only once (63%) by the partner. In general patients of the partner where presented twice or three times: the first time their diagnostic plan, the second time the therapeutic plan and sometimes surgical results the third time (55 out of 177 – 31%). Only one patient was discussed four times; five patients on the partner's list were not discussed at the first opportunity because imaging was not complete.

There were significant differences ($p < .001$) in the cases presented by the centre and the partner regarding tumour localization, histology and tumour stage (Table 2): the partner presented more cases with infections that were initially suspected malignancy, T1-stage patients or non-complex cases. Not all specialisms were present during all the videoconference meetings. In 61% of the 18 videoconferences both teams were complete; the centre team was not complete in 22% (n=4) and, in 17% (n=3), the partner team was not complete. The centre's ENT department was represented in most meetings by a fellow training to be a head and neck surgeon. The centre presented on average 5.2 (SD 2.4) cases per videoconference, the partner presented on average 13.5 (SD 3.9) cases.

Recommendations given

Recommendations were given in 8 of the 336 cases presented (2%; 95% confidence interval: 1 to 5%) relating to 8 of the 259 patients (3%; 95% confidence interval: 1 to 6%). In the pilot study we found that 'some kind of advice' was given in 20% of the cases.

Of these recommendations, five were major and three minor (Table 3). Four recommendations concerned diagnostic plans, and four treatment plans. On three of the eight occasions when a recommendation was given, the centre's team was incomplete with one of the three specialisms absent. Seven of the eight recommendations were given by OMS specialists, and five of the eight were related to ENT patients. Seven of the eight instances occurred on a patient's first presentation, and the other one during a second presentation although, in this case, the imaging had not been complete the first time. In general, recommendations were given related to the more complex cases, but not necessarily patients with comorbidity or those with more advanced tumours.

For peer review only - http://bmjopen.bmj.com/site/about/guidelines.xhtml

Table 3 Recommendation and its specifics

No	Recommen-dation	Who	To whom	Team complete?	Recommen-dation (short)	Change impact, diagnosis or treatment phase	Patient status (ICD-code, TNM-classification, histology; case complexity, guideline used and comorbidity)					
							ICD	TNM	histology	com-plex?	guide-line?	comor-bid?
1	2016G10-1 28-09-2016	OMS partner	ENT centre	yes	give patient choice of expectative policy	major, treatment	C44	T2N0M0	SCC	yes	no	yes
2	2016L14-1 28-09-2016	OMS centre	OMS partner	yes	ultrasound guided biopsy	minor, diagnosis	-	-	maligne lympho-ma	no	yes	no
3	2016G32-1 26-10-2016	OMS partner	OMS centre	centre not	use methotrexate to identify malignancy	minor, treatment	C00	T1N0M0	SCC	yes	yes	no
4	2016G39-1 23-11-2016	OMS partner	ENT centre	yes	change surgery approach to retain functionality	major, treatment	C00	T2N0M0	adenoid cystic carcinoma	yes	no	no
5	2016G40-1 23-11-2016	OMS partner	ENT centre	yes	try PDT	major, treatment	C01	T4aN0M0	SCC	yes	no	no
6	2016G51-1 14-12-2016	OMS partner	ENT centre	centre not	consult Ophthalmo-logy	major, diagnosis	C44	T2N0M0	BCC eye corner	yes	no	yes
7	2016L90-2 14-12-2016	OMS centre	ENT partner	centre not	new biopsy	major, diagnosis	C31	T3NxM0	Melan.	yes	yes	yes
8	2017L123-1 04-01-2017	RT centre	OMS partner	yes	add MRI	minor, diagnosis	C07	T1N0M0	SCC	yes	yes	no

Squamous cell carcimoma (SCC), BCC = Basal cell carcinoma, Melan. = Melanoma; MRI = Magnetic Resonance Imaging; PDT = Photo Dynamic Therapy

For peer review only - http://bmjopen.bmj.com/site/about/guidelines.xhtml

Qualitative analysis – specialist interviews

From the transcripts of the six interviews, 107 quotes were registered. During the coding procedure, items were placed in a coding tree with relevance to the primary research question (recommendations given) and the secondary research question (added value as described in terms of perceived benefits and drawbacks) by the researcher in consultation with the coders. For each major theme, minor themes were derived from the researcher's field notes. In total 282 scores were given (Table 4). In several instances the quotes were scored differently although the inter-coder agreement was acceptable given the possible 37 codes to choose from.

Codes were judged as being a benefit or a drawback. Benefits were more frequently mentioned by specialists of the partner, and the drawbacks more frequently by specialists of the centre. But the majority of given codes by the coders (of the centre and of the partner) had a positive connotation for the videoconferencing (Table 4).

For peer review only

For peer review only - http://bmjopen.bmj.com/site/about/guidelines.xhtml

Table 4 Coding tree evaluation videoconferencing

Coding tree			Pos?	Code	Code description	Part-ner	Centre	Total
Videoconferencing	Recom-menda-tion	Nuance	+	22	videoconferencing is mostly ‘inter-collegial consultation’	3	3	6
			+	14	recommendations are nuances, not a totally different medical policy or diagnostic/treatment plan for a specific patient	7	10	17
		Follow-up traceable?	+	6	suggestions are taken from others	1	2	3
			+	20	there is no patient-level evaluation on the implementation of medical policies agreed, question of trust	3	2	5
			-	34	sometimes decisions are already taken in relation to continuity of treatment	1	1	2
		Aligning	+	1	fine-tuning or aligning medical policies	10	10	20
			+	9	continue routine cases discussion to prevent deviation from medical policies	2	2	4
		Knowledge	0	32	besides videoconferencing also bilateral consultation via telephone	4	1	5
			+	37	keep ‘know how’ with routine cases	1	2	3
	Added Value?	Videoconfe-rence session	+	8	added value for complex cases vs. routine cases	21	24	45
			-	15	little added value	8	1	9
			0	27	discuss radio-therapeutic scheme	2	2	4
			-	29	non-complex cases or ‘formalities’ are communicated because it is mandatory, no added value	7	1	8
			+	30	recommendation given to own discipline	5	1	6
		Team completeness	+	4	presence of all three disciplines is essential	3	4	7
			+	11	expertise (good) of physician is important	5	3	8
			0	23	add presence of medical oncology discipline as expertise	2	2	4
	Collabo-ration	Communi-cation	0	2	working together requires communication	8	2	10
			+	10	at both locations working methods are comparable through videoconferencing	5	2	7
			-	19	initially it was good to consult, added value decreased because teams have grown towards each other	1	1	2
		Trust	+	5	respectful collaboration	3	7	10
			+	7	mutual trust	4	5	9
			+	13	important to know the partner, not only via videoconferencing; good for cohesion	8	7	15
		Expertise	-	18	centre member does not think videoconferencing necessary, because partner should be trusted as such	2	4	6
			+	26	expertise and new developments from centre to partner	2	2	4
		DHCI	0	21	videoconferencing between centre and partner is a national agreement or policy	2	3	5

For peer review only

For peer review only - http://bmjopen.bmj.com/site/about/guidelines.xhtml

Coding tree			Pos?	Code	Code description	Part-ner	Centre	Total
		requirement	-	31	the national policy – to discuss all cases including routine cases – between centre and partner is perceived as out-dated	7	2	9
	Plan-ning	Logistics	-	16	stressful, considering other videoconferences	3	6	9
			0	17	integrate videoconferencing in the hospital's videoconference for centre and partner	5	7	12
		Preparation	-	12	improve format of patient presentation	1	1	2
			+	24	good preparation is important	5	4	9
		Commitments	+	25	starting and stopping the videoconferencing on time is important	4	1	5
			-	33	possibly cancel videoconferencing when nothing to discuss	1	1	2
		Equipment	+	3	technique always flawless	1	1	2
			-	35	sometimes videoconferencing did not take place due to technical malfunction	1	1	2
			-	36	placement of monitor in the room hinders colleagues and hampers interaction	2	2	4
Scientific Research			0	28	bias through research setting because researcher as observer is present (Hawthorne effect)	1	1	2
					Total quotes	151	131	282

This coding tree has major and minor themes that were derived from the primary research question (recommendations given), the secondary research question (added value as described in benefits and drawbacks perceived) and minor themes derived from researcher's field notes. One code was related to the research situation.

'Pos?' refers to the question: has this code a positive connotation or benefit? + = yes, 185 scores; 0 = neither positive nor negative, 42 scores; - = no, 55 scores.

The amount of codes given is given for the partner, the centre and in total. DHCI is short for Dutch Health Care Inspectorate.

For peer review only

For peer review only - http://bmjopen.bmj.com/site/about/guidelines.xhtml

Six main items were important in the view of the specialists that were interviewed, each illustrated with one or two quotes in italic.

1. The videoconference adds value when discussing complex cases, through assisting in fine tuning and aligning medical procedures (code 1, 20x);

A patient is presented about which the own team had some discussion, that can be discussed with the partner. In that manner you get a confirmation or advice to change your policy. This advice can be given by the same specialism, but also by other members of the Head and Neck Oncology team (ENT).

2. Communication is essential for cooperation between the teams (code 2, 10x), furthermore it is important to know the partner well, not only via videoconferencing (code 13, 15x) and to interact respectfully (code 5, 10x) with mutual trust (code 7, 9x).

The most important feature of the videoconferencing is to communicate with each other on substantive medical matters, to be on speaking terms, and to know each other (RT).

During the videoconferencing we respect each other, we listen to each other and we are open to each other's additional comments. We trust each other as partners (OMS).

3. Recommendations are suggested alternatives on diagnostic modalities and treatment plans for specific patients (code 14, 17x).

The videoconference has the character of a collegial discussion, in which in collaboration the best diagnostic or treatment plan for your patient is reached. Confirmation on your policy adds value too (OMS).

4. For non-complex cases that fall within guideline for treatment, the videoconference meeting adds little value as for changes in medical content, it can even irritate the participants in such cases (code 15, 9x).

The videoconference sometimes changes a policy for an individual patient. The videoconference is not the meeting where new policies or guidelines are developed (RT).

5. There is a wish to integrate the videoconference with the site multidisciplinary meeting in both hospitals, the centre and the partner (code 17, 12x).

Integration of the two local multidisciplinary meetings with the videoconference could be valuable (ENT).

For peer review only - http://bmjopen.bmj.com/site/about/guidelines.xhtml

For peer review only

6. The DHCI requirement (discuss all the partner's cases with the centre) is not felt to add value, but rather seen as old-fashioned or out-dated (code 29, 8x).

It is better to prepare at a high level and discuss, than to present all the patients and deal with each one briefly. Mutual preparation on special request could have added value, for example a literature search on a complex osteosarcoma case (OMS).

DISCUSSION

Our results show that the added value of the weekly multidisciplinary videoconference between the head and neck cancer centre and the preferred partner hospital was small given the few recommendations made on the initial diagnostic and/or therapeutic treatment plan (in only 2% of the 336 cases presented). Nevertheless, the specialists from both sites recognized the importance of keeping their medical viewpoints aligned through this type of communication. Whenever discussing complex cases in which a major change was recommended (in 5 of the 8 recommendations), for example to change the surgical approach to save functionality of organs or tissue, the recommended change in treatment had a large impact for that patient (Table 3).

Contrary to our findings from the pilot study, where advice was offered in 20% of the presented cases and on which the estimated sample size was based, the actual 2% recommendations is much lower. Although it is difficult to explain this difference, we think that the pilot served mainly as a feasibility check, that helped us to define our research questions and to operationalize the definitions. Other factors may also have played a role in the difference of outcomes between the pilot and the actual study. Firstly, the long-lasting cooperation between the centre and the partner had led to a high level of alignment on diagnostic and therapeutic 'strategies' or medical viewpoints. Secondly, the participants were not blinded for the research question. Thus awareness of being part of an experiment may have led to a drive to perform well and to present the patients with an optimal diagnostic and treatment plan, also called 'the Hawthorn effect'. Often the teams mentioned that the other team was asked to give collegial advice and therefore a suggestion was not always seen as a recommendation. Thirdly, some patient cases were only presented as interesting to discuss. Finally, during the pilot study the advice given was not assessed for its impact.

For peer review only - http://bmjopen.bmj.com/site/about/guidelines.xhtml

In the qualitative part of this study the interviews on the benefits and drawbacks of videoconferencing revealed that medical specialists perceive added value in discussing complex cases in a collegiate consultation, because another team can offer a fresh perspective by hearing the case ‘as new’. Although remarks were often about nuances, the confirmation on the chosen policy by the other team was experienced as helpful. This view is supported in literature where medical specialists found videoconferencing useful in at least one aspect of their practice[9].

An important condition to communicate through videoconference is that participants should know each other from meetings with physical attendance, to support mutual trust and respect as the basis for the cooperation. Two studies support the finding in their conclusions, stating that collaboration and cooperation improves when each discipline understands each other’s roles and that specialties working together for a long time do not need many words to come to a decision[15, 25].

The videoconference meeting can be used to introduce and discuss new developments, protocols and guidelines leading to comparable quality of care in both locations. Two studies found that a comprehensive cancer centre team working together over videoconferencing with a peripheral hospital team align their treatment plans: discussing radiotherapy treatment plans changes are major and minor, 7 % of cases and 21% respectively[14] and speed up follow-up appointments[13].

The weekly multidisciplinary videoconference meeting differs from the local multidisciplinary meeting: during videoconferencing complex cases are discussed with a second ‘expert team’ of head and neck oncology specialists. The patients treated by the centre and by the partner are comparable, although diagnostics and treatment might differ slightly[26]. In our study the significant differences in terms of tumour localization, cell type and tumour stage seen in the presented cases are a consequence of ‘the DHCI requirement’ (all patients treated by the ‘preferred partner’ should be discussed in a multidisciplinary patient meeting with the centre), whereas the ‘centre’ could decide which of its patients would make an interesting case for discussing with their partner. As a consequence, the partner presents 3 to 4 times as many patients as the centre. One third of these (31%) reappear in the subsequent videoconferences, checking: extra diagnostic information, the treatment plan and the need for adjuvant therapy. Most of this is seen as a ‘formality (checking compliance to guidelines)’. The data from the interviews suggest that especially complex patient cases would benefit from inter

For peer review only - http://bmjopen.bmj.com/site/about/guidelines.xhtml

collegial consultation via videoconferencing. If the teams were not obliged to discuss so many straightforward cases, they could use the time saved to prepare and discuss complex cases in greater depth (Results, finding 6, quote OMS)[27].

The perceived value of the videoconference might be influenced by the expertise of a specialist. The recommendations given during the evaluation period were mostly given to ENT by an OMS oncologist who had considerably more clinical experience than his opposing fellow, and was also one of the instigators of the videoconference. It could be that with a marked difference in experience, recommendations are given and accepted more easily[11]. This way videoconferencing supports specialists getting experience with presenting complex oncology patient cases and with decision making in teams[5, 15].

In this study, we evaluated in depth the added value of a multidisciplinary videoconference meeting between one oncology centre and its preferred partner. In line with other studies[28, 29], this study shows that, in addition to a quantitative result (number of recommendations), it is important to reflect on the situation through an interview process (qualitative results) before starting to implement improvements. What our interviews showed is that the specialists at both the centre and the partner support the idea of sustainable cooperation, but they do not support the view implicit in the DHCI requirement that the centre should act as means of quality control for the partner[30]. Our findings on videoconferencing are supported by others in terms of the positive results on teams working together. More studies have shown that more research is needed to understand the effects of videoconferencing on patient outcomes such as finance including resource usage[31, 32], what fields of specialisms could benefit from the medium[25], participant satisfaction[33], throughput times[34] and self-management for patients[35].

In summary, based on our findings, we believe that the DHCI requirement (the partner should discuss all patients with the centre) is unnecessary in the case of routine patients, since it does not add value to the quality of their treatment. It is more useful to spend time on complex cases in greater detail. We propose the following measures that will add value to the weekly videoconference:

1. All the participating medical specialists should be granted freedom to select only complex or interesting cases that could serve to keep medical procedures aligned.

For peer review only - http://bmjopen.bmj.com/site/about/guidelines.xhtml

2. The partner should not be obliged to present cases seen as 'formalities' since this does not add value.
3. The videoconference should be organized as an integral part of the partners' multidisciplinary meetings and not as a separate weekly meeting.

Based on our findings on the added value of the multidisciplinary videoconference between the head and neck centre and its partner organization, and our suggestions for improvements in this activity, we would advise the DHNS, along with healthcare policymakers, to reconsider making the DHCI requirement.

In our study we found that there are clinical and practical implications on how and when to start with videoconferencing instead of meetings with physical attendance. Videoconferencing must be seen as a supportive medium for communication within a sustainable collaboration of parties that understand each other's roles and work with guidelines or protocols.

Participants of a videoconference should:

1. Know each other, and meet face-to-face on a regular basis.
2. Respect each other as 'expert / knowing' colleague and know each other's role in the multidisciplinary treatment of patients.
3. Trust each other in follow-up of changes to diagnostic and treatment plans.

In view of the above mentioned implications we would not recommend starting with videoconferencing for multidisciplinary meetings if a majority of participants do not know each other.

CONCLUSIONS

The videoconferencing has added value in the cooperation and in the care pathways management. Centre and partner align their medical policies when interpreting national multidisciplinary guidelines. The videoconference meeting provides a means to discuss complex cases with another 'expert team' with a fresh perspective to reach an optimal decision regarding diagnostic and treatment plans. Conversely, discussing non-complex cases is seen as an unnecessary burden, and the DHCI requirement to discuss all the partner's cases as out-dated.

LIST OF ABBREVIATIONS

For peer review only - http://bmjopen.bmj.com/site/about/guidelines.xhtml

CRF	Case Report Form
DMD	Doctor of Dental Medicine
DDS	Doctor of Dental Surgery
DHNS	Dutch Head and Neck Society
DHCI	Dutch Health Care Inspectorate
ENT	Ear, Nose and Throat
ICD(O)	International Classification of Diseases (of Oncology)
MCL	Medical Centre Leeuwarden
MeSH	Medical Subject Headings
OMS	Oral and Maxillofacial Surgery
RT	Radiotherapy
SPSS	Statistical Package for Social Sciences
UMCG	University Medical Center Groningen

DECLARATIONS

Ethics approval and consent to participate

This prospective observational study on decision-making analysis was checked by the Medical Ethics Review Board of the UMCG (2016, ref. M16.194909), the Netherlands. They concluded that the study is not a 'clinical research study with human subjects' as meant in the Medical Research Involving Human Subject Act (WMO). Informed consent was not required. The Dutch law requires also a privacy statement from the partner in the study, the Medical Centre Leeuwarden (2016, nWMO 187).

Data sharing statement

Datasets will be available from the corresponding author on request.

Consent for publication

Not applicable.

Competing interests: No authors have competing interests.

Funding: This research received no specific grant from any funding agency in the public, commercial or not-for-profit sectors.

Authors' Contribution

For peer review only

LvH was involved in the study design and concept; carried out the study; performed the statistical analysis and the analysis and interpretation of the data; and drafted the manuscript. PD, KA, JdV and JR, the supervisor, were involved in the study design and concept, analysis and interpretation of the data, and revision of the manuscript. JdV and JR were involved in the coding of the interview quotations, together with LvH. GH, JvdH, KvdL and OW were involved in the acquisition of the data and the revision of the manuscript.

All authors read and approved the final manuscript.

Acknowledgements

This research was sponsored by the University Medical Centre Groningen.

Authors' information

The University Medical Center Groningen is developing patient centred care pathways for diverse patient groups including laws and regulations for quality and patient safety. LvH, JR are working in cooperation with KA of the Centre of Expertise Healthwise to research care pathway implementation in the Comprehensive Cancer Center Groningen and to develop quality and safety indicators, i.e. process indicators that predict performance of care pathways and sustainable patient outcome.

REFERENCES

1 Ouwens M, Hermens R, Hulscher M, et al. Development of indicators for patient-centred cancer care. *Support Care Cancer* 2010;18:121-30 doi:10.1007/s00520-009-0638-y; 10.1007/s00520-009-0638-y.

2 Policy head and neck cancer care 2013 (in Dutch). Available at: http://www.nwhht.nl/organisatie/missie.

3 Halmos GB, Bras L, Siesling S, et al. Age-specific incidence and treatment patterns of head and neck cancer in the Netherlands-A cohort study. *Clin Otolaryngol* 2018;43:317-24 doi:10.1111/coa.12991 [doi].

4 Fleissig A, Jenkins V, Catt S, et al. Multidisciplinary teams in cancer care: are they effective in the UK?. *Lancet Oncol* 2006;7:935-43 doi:S1470-2045(06)70940-8 [pii].

5 Ruhstaller T, Roe H, Thurlimann B, et al. The multidisciplinary meeting: An indispensable aid to communication between different specialities. *Eur J Cancer* 2006;42:2459-62 doi:S0959-8049(06)00555-7 [pii].

For peer review only - http://bmjopen.bmj.com/site/about/guidelines.xhtml

6 Ouwens MM, Hermens RR, Hulscher MM, et al. Impact of an integrated care program for patients with head and neck cancer on the quality of care. *Head Neck* 2009;31:902-10 doi:10.1002/hed.21041; 10.1002/hed.21041.

7 Dutch National Cancer Control Programme. Progress Report on Cancer Control in the Netherlands, 2005-2010 (Dutch NCCP, Nationaal programma kankerbestrijding). 2010.

8 Cijfers over kanker / Figures on cancer. Available at: http://www.cijfersoverkanker.nl. Accessed May, 6, 2017.

9 Norum J, Jordhoy MS. A university oncology department and a remote palliative care unit linked together by email and videoconferencing. *J Telemed Telecare* 2006;12:92-6 doi:10.1258/135763306776084374 [doi].

10 Bydder S, Nowak A, Marion K, et al. The impact of case discussion at a multidisciplinary team meeting on the treatment and survival of patients with inoperable non-small cell lung cancer. *Intern Med J* 2009;39:838-41 doi:10.1111/j.1445-5994.2009.02019.x [doi].

11 Slavova-Azmanova NS, Johnson CE, Platell C, et al. Peer review of cancer multidisciplinary teams: is it acceptable in Australia?. *Med J Aust* 2015;202:144-7 doi:10.5694/mja14.00768 [pii].

12 van Huizen LS, Dijkstra PU, van der Laan BFAM, et al. Multidisciplinary first-day consultation accelerates diagnostic procedures and throughput times of patients in a head-and-neck cancer care pathway, a mixed method study. *BMC Health Serv Res* 2018;18:820,018-3637-1 doi:10.1186/s12913-018-3637-1 [doi].

13 Farris G, Sircar M, Bortinger J, et al. Extension for Community Healthcare Outcomes-Care Transitions: Enhancing Geriatric Care Transitions Through a Multidisciplinary Videoconference. *J Am Geriatr Soc* 2017;65:598-602 doi:10.1111/jgs.14690 [doi].

14 Seeber A, Mitterer M, Gunsilius E, et al. Feasibility of a multdisciplinary lung cancer videoconference between a peripheral hospital and a comprehensive cancer centre. *Oncology* 2013;84:186-90 doi:10.1159/000345314 [doi].

15 Gagliardi A, Smith A, Goel V, et al. Feasibility study of multidisciplinary oncology rounds by videoconference for surgeons in remote locales. *BMC Med Inform Decis Mak* 2003;3:7,6947-3-7. Epub 2003 Jun 19 doi:10.1186/1472-6947-3-7 [doi].

16 Charmaz K. Contructing Grounded Theory. A Practical Guide through Qualitative Analysis. London: Sage Publications 2006.

17 Plochg T, Juttman RE, Klazinga NS, et al. Handbook health research (Handboek gezondheidszorgonderzoek). Houten: Bohn Stafleu van Loghum 2007.

18 O'Brien BC, Harris IB, Beckman TJ, et al. Standards for reporting qualitative research: a synthesis of recommendations. *Acad Med* 2014;89:1245-51 doi:10.1097/ACM.0000000000000388 [doi].

19 Kallogjeri D, Piccirillo JF, Spitznagel EL,Jr, et al. Comparison of Scoring Methods for ACE-27: Simpler Is Better. *J Geriatr Oncol* 2012;3:238-45 doi:10.1016/j.jgo.2012.01.006 [doi].

20 Paleri V, Wight RG, Silver CE, et al. Comorbidity in head and neck cancer: a critical appraisal and recommendations for practice. *Oral Oncol* 2010;46:712-9 doi:10.1016/j.oraloncology.2010.07.008 [doi].

For peer review only - http://bmjopen.bmj.com/site/about/guidelines.xhtml

For peer review only

21 Tong A, Sainsbury P, Craig J. Consolidated criteria for reporting qualitative research (COREQ): a 32-item checklist for interviews and focus groups. *Int J Qual Health Care* 2007;19:349-57 doi:mzm042 [pii].

22 Gioia DA, Gorley KG, Hamilton AL. Seeking Quality Rigor in Inductive Research: Notes on the Gioia Methodology. *Organizational Research Methods* 2012;16:15,16-31 doi:10.1177/1094428112452151.

23 Birt L, Scott S, Cavers D, et al. Member Checking: A Tool to Enhance Trustworthiness or Merely a Nod to Validation?. *Qual Health Res* 2016 doi:1049732316654870 [pii].

24 Vlaams Kankerregistratienetwerk. International Classification of Diseases for Oncology, third edition, updates (in Dutch): Vlaams Kankerregistratienetwerk 2011:240.

25 Stevens G, Loh J, Kolbe J, et al. Comparison of recommendations for radiotherapy from two contemporaneous thoracic multidisciplinary meeting formats: co-located and video conference. *Intern Med J* 2012;42:1213-8 doi:10.1111/j.1445-5994.2012.02817.x [doi].

26 de Ridder M, Balm AJM, Baatenburg de Jong RJ, et al. Variation in head and neck cancer care in the Netherlands: A retrospective cohort evaluation of incidence, treatment and outcome. *Eur J Surg Oncol* 2017;43:1494-502 doi:S0748-7983(17)30364-5 [pii].

27 Xylinas E, Roupret M, Kluth L, et al. Collaborative research networks as a platform for virtual multidisciplinary, international approach to managing difficult clinical cases: an example from the Upper Tract Urothelial Carcinoma Collaboration. *Eur Urol* 2012;62:943-5 doi:10.1016/j.eururo.2012.08.022 [doi].

28 Helck A, Matzko M, Trumm CG, et al. Interdisciplinary expert consultation via a teleradiology platform--influence on therapeutic decision-making and patient referral rates to an academic tertiary care center. *Rofo* 2009;181:1180-4 doi:10.1055/s-0028-1109733 [doi].

29 Ottevanger N, Hilbink M, Weenk M, et al. Oncologic multidisciplinary team meetings: evaluation of quality criteria. *J Eval Clin Pract* 2013;19:1035-43 doi:10.1111/jep.12022 [doi].

30 Stoffels AR. Cooperation among medical specialists: pain or gain? 2008.

31 Sezeur A, Degramont A, Touboul E, et al. Teleconsultation before chemotherapy for recently operated on patients. *Am J Surg* 2001;182:49-51 doi:S0002-9610(01)00662-6 [pii].

32 Kunkler IH, Fielding RG, Brebner J, et al. A comprehensive approach for evaluating telemedicine-delivered multidisciplinary breast cancer meetings in southern Scotland. *J Telemed Telecare* 2005;11 Suppl 1:71-3 doi:10.1258/1357633054461804 [doi].

33 Careau E, Dussault J, Vincent C. Development of interprofessional care plans for spinal cord injury clients through videoconferencing. *J Interprof Care* 2010;24:115-8 doi:10.3109/13561820902881627 [doi].

34 Fitzpatrick D, Grabarz D, Wang L, et al. How effective is a virtual consultation process in facilitating multidisciplinary decision-making for malignant epidural spinal cord compression?. *Int J Radiat Oncol Biol Phys* 2012;84:e167-72 doi:10.1016/j.ijrobp.2012.03.057 [doi].

For peer review only

For peer review only - http://bmjopen.bmj.com/site/about/guidelines.xhtml

35 Tokuda L, Lorenzo L, Theriault A, et al. The utilization of video-conference shared medical appointments in rural diabetes care. *Int J Med Inform* 2016;93:34-41 doi:10.1016/j.ijmedinf.2016.05.007 [doi].

For peer review only

For peer review only - http://bmjopen.bmj.com/site/about/guidelines.xhtml

COREQ checklist

**Title of study**	Does the multidisciplinary videoconferencing between a head and neck cancer centre and its preferred partner add value to their patient care and to their decision making? A mixed method evaluation.		
**Domain**	Details of Items	**Answer**	**Location in manuscript**
**Domain 1: research team and reflexivity**			
Personal Characteristics [5 items]			
1	Interviewer (Which author/s conducted the interview or focus group?)	LvH	Methods
2	Credentials (What were the researcher's credentials? E.g. PhD, MD)	MSc	Author's information
3	Occupation (What was their occupation at the time of the study?)	Sr. Consultant Quality and Patient Safety	Author's information
4	Gender	Female	Author's information
5	Experience & Training	LvH is consultant & trainer and is experienced using diverse qualitative analysis and improvement methods.	CV in Research file
Relationship with participants [3 items]			
6	Prior Relationship (Was a relationship established prior to study commencement?)	Researcher participates in the research group of the Head & Neck Oncology care pathway and was attending videoconferencing before start of this study.	CV in Research file
7	Participant knowledge of interviewer (e.g. personal goals, reasons for doing the research)	As a researcher, but not personal.	-
8	Interviewer characteristics (e.g. Bias, assumptions, reasons and interests in the research topic)	Researcher supports as Quality coordinator the University Medical Centre for many patient flows, including the Head & Neck Oncology care pathway .	CV in Research file
**Domain 2:** study design			
Theoretical framework [1 item]			
9	Methodological orientation and Theory (What methodological orientation was stated to underpin the study? e.g. grounded theory, discourse analysis, ethnography, phenomenology, content analysis)	Content analysis	Methods
Participant selection [4 items]			
10	Sampling (e.g. purposive, convenience, consecutive, snowball)	Consecutive	Methods
11	Method of approach (e.g. face-to-face, telephone, mail, email)	Face-to-face	Methods

For peer review only

12	Sample size (How many participants were in the study?)	3 specialists of centre and partner team.	Methods
13	Non-participation (How many people refused to participate or dropped out? Reasons?)	None	Methods
Setting [3 items]			
14	Setting of data collection (Where was the data collected? e.g. home, clinic, workplace)	Data of interviews were collected in the clinic	Methods
15	Presence of non-participants (Was anyone else present besides the participants and researchers?)	No	
16	Description of sample (What are the important characteristics of the sample? e.g. demographic data, date)	Patients & tumour characteristics; participating specialists	Methods, results, table 2; methods, results.
Data collection [7 items]			
17	Interview guide (Were questions, prompts, guides provided by the authors? Was it pilot tested?)	Interviews were semi-structured using an interview guide schedule; follow up questions were allowed. Participants were also asked to point out improvements.	Methods, textbox 1.
18	Repeat interviews (Were repeat interviews carried out? If yes, how many?)	No	Methods
19	Audio/visual recording (Did the research use audio or visual recording to collect the data?**)**	The semi-structured interviews were audio recorded using a smartphone.	Methods
20	Field notes (Were field notes made during and/or after the interview or focus group?**)**	Researcher observations were registered during the videoconferencing registration period and used for set-up of questions in the semi-structured interview phase and for development of minor themes in the coding tree.	Methods
21	Duration of interviews	25 to 40 minutes.	Methods
22	Data saturation (Was data saturation discussed?)	Pre-selected participants were all interviewed: 3 specialists form each team representing all medical specialisms in the videoconferencing.	Methods
23	Transcripts returned (Were transcripts returned to participants for comment and/or correction?)	Transcripts were returned to the	Methods

For peer review only

		participants with quotes. To check results the member check method (Synthesized Member Checking, Birt et al. 2016) was used.	
**Domain 3:** analysis and findings			
Data analysis – 5 items]			
24	Number of data coders	One made codes, a second used the codes	Methods
25	Description of coding tree	Yes	Methods, results table 4
26	Derivation of themes (Were themes identified in advance or derived from the data?)	Major themes were derived from the research questions and minor themes were derived from researcher's field notes during analysis.	Methods, results
27	Software (to manage the data)	Word, Excel, SPSS	Methods
28	Participant checking (Did participants provide feedback on the findings?)	To check results the member check method (Synthesized Member Checking, Birt et al. 2016) was used.	Methods
Reporting [4 items]			
29	Quotations presented (Were participant quotations presented to illustrate themes/ findings? Was each quotation identified? E.g. participant number)	Quotes are provided as illustration of main findings and are attributed to particular type of participants.	Methods, results
30	Data and findings consistent (Was there consistency between the data presented and the findings?)	Yes, the same patterns were observed with all participants.	Results, discussion
31	Clarity of major themes (Were *major* themes clearly presented in the findings?)	Coding tree (see underneath, last row)	Methods, results, table 4
32	Clarity of minor themes (Is there a description of diverse cases or discussion of minor themes?)	Yes	Results, discussion
Coding tree contains the following *Major* and Minor themes within a major theme:<br>*Recommendation*: Nuance, Follow-up traceable?, Aligning, Knowledge<br>*Added Value?:* Videoconference session, Team completeness,<br>*Collaboration:* Communication, Trust, Expertise, Dutch Health Care Inspectorate (DHCI) requirement<br>*Planning*: Logistics, Preparation, Commitments. Equipment<br>*Scientific Research*			

For peer review only

BMJ Open

Does multidisciplinary videoconferencing between a head and neck cancer centre and its partner hospital add value to their patient care and decision making? A mixed method evaluation

Journal:	*BMJ Open*
Manuscript ID	bmjopen-2018-028609.R1
Article Type:	Original research
Date Submitted by the Author:	26-Jul-2019

Complete List of Authors:	van Huizen, Lidia; University of Groningen, University Medical Center Groningen, Quality and Patient Safety; Oral and Maxillofacial Surgery<br>Dijkstra, Pieter; University of Groningen, University Medical Center Groningen, Centre for Rehabilitation<br>Halmos, Gyorgy; University of Groningen, University Medical Center Groningen, Ear, Nose and Throat<br>van den Hoek, Johanna; University of Groningen, University Medical Center Groningen, Radiotherapy<br>van der Laan, Klaas; Medical Center Leeuwarden, Ear, Nose and Throat<br>Wijers, Oda; Radiotherapeutic Institute Friesland<br>Ahaus, Kees; University of Groningen, Faculty of Economics and Business, Operations; Erasmus Universiteit Rotterdam, Erasmus School of Health Policy & Management<br>de Visscher, Jan; Medical Center Leeuwarden, Oral and Maxillofacial Surgery; Free University Medical Center, Oral and Maxillofacial Surgery / Oral Pathology<br>Roodenburg, Jan; University of Groningen, University Medical Center Groningen, Oral and Maxillofacial Surgery

**Primary Subject Heading**:	Oncology
Secondary Subject Heading:	Health policy, Qualitative research
Keywords:	Videoconferencing, Collaborating teams, Mixed method study, Added value, Head & neck tumours < ONCOLOGY, Multidisciplinary team meetings (MDT)

SCHOLARONE™
Manuscripts

For peer review only - http://bmjopen.bmj.com/site/about/guidelines.xhtml

Does multidisciplinary videoconferencing between a head-and-neck cancer centre and its partner hospital add value to their patient care and decision-making? A mixed method evaluation

Lidia S. van Huizen (LvH), MSc[1,2], corresponding author, l.s.van.huizen@umcg.nl, +31 50 361 8035

Pieter U. Dijkstra (PD), PT, PhD[1,3], p.u.dijkstra@umcg.nl

Gyorgy B. Halmos (GH), MD, PhD[4], g.b.halmos@umcg.nl

Johanna G.M. van den Hoek (JvdH), MD[5], j.g.m.van.den.hoek@umcg.nl

Klaas T. van der Laan (KvdL), MD, PhD[6], author deceased May 4th 2019

Oda B. Wijers (OW), MD, PhD[7], o.b.wijers@skf-rif.nl

Kees (C.)T. B. Ahaus (KA), PhD[8, 9], ahaus@eshpm.eur.nl

Jan G.A.M. de Visscher (JdV), DMD, MD, PhD[10,11], j.de.visscher@znb.nl

Jan L.N. Roodenburg (JR), DDS, MD, PhD[1], j.l.n.roodenburg@umcg.nl

[1] University of Groningen, University Medical Center Groningen, Department of Oral and Maxillofacial Surgery, Groningen, The Netherlands

[2] University of Groningen, University Medical Center Groningen, Department of Quality and Patient Safety, Groningen, The Netherlands

[3] University of Groningen, University Medical Center Groningen, Centre for Rehabilitation, Groningen, The Netherlands

[4] University of Groningen, University Medical Center Groningen, Department of Ear, Nose & Throat / Head and Neck Surgery, Groningen, The Netherlands

[5] University of Groningen, University Medical Center Groningen, Department of Radiotherapy, Groningen, The Netherlands

[6] Medical Centre Leeuwarden, Department of Ear, Nose & Throat, Leeuwarden, The Netherlands

[7] Radiotherapeutic Institute Friesland, Leeuwarden, the Netherlands

[8] University of Groningen, Faculty of Economics and Business, Centre of Expertise Healthwise, University Medical Center Groningen, Groningen, The Netherlands

For peer review only

For peer review only - http://bmjopen.bmj.com/site/about/guidelines.xhtml

[9] Erasmus University, Erasmus School of Health Policy & Management, Rotterdam, The Netherlands

[10] Medical Centre Leeuwarden, Department of Oral and Maxillofacial Surgery, Leeuwarden, The Netherlands

[11] Free University Medical Center, Department of Oral and Maxillofacial Surgery / Oral Pathology, Amsterdam, The Netherlands

ABSTRACT

Objectives

Given the difficulties in diagnosing and treating head-and-neck cancer, care is centralized in the Netherlands in eight head-and-neck cancer centres and six satellite regional hospitals as preferred partners. A requirement is that all patients of the partner should be discussed in a multidisciplinary team meeting (MDT) with the head-and-neck centre as part of a Dutch health policy rule. In this mixed method study, we evaluate the value that the video-conferenced MDT adds to the MDTs in the care pathway, quantitative regarding recommendations given and qualitative in terms of benefits for the teams and the patient.

Design

A sequential mixed method study

Setting

One oncology centre and its partner in the Northern part of the Netherlands

Participants

Head-and-neck cancer specialists presenting patient cases during video-conferenced MDT over a period of six months. Semi-structured interviews held with six medical specialists, three from the centre and three from the partner.

Primary and secondary outcome measures

Percentage of cases in which recommendations were given on diagnostic and/or therapeutic plans during video-conferenced MDT.

Results

For peer review only

For peer review only - http://bmjopen.bmj.com/site/about/guidelines.xhtml

In eight of the 336 patient cases presented (2%), specialists offered recommendations to the collaborating team (3 given from centre to partner and 5 from partner to centre). Recommendations mainly consisted of alternative diagnostic modalities or treatment plans for a specific patient. Interviews revealed that specialists perceive added value in discussing complex cases because the other team offered a fresh perspective by hearing the case ‘as new’. The teams recognize the importance of keeping their medical viewpoints aligned, but the requirement (that the partner should discuss all patients) was seen as out-dated.

Conclusions

The added value of the video-conferenced MDT is small considering patient care, but the specialists recognized that it is important to keep their medical viewpoints aligned and that their patients benefit from the discussions on complex cases.

Keywords

Videoconferencing (MeSH term), head-and-neck cancer, collaborating teams, multidisciplinary team meetings (MDT), added value, mixed method study

Strengths and limitations of this study

- The study evaluates in depth the video-conferenced MDT between the centre and the partner in the head-and-neck oncology care pathway and refocuses on benefits and drawbacks (strength)
- Participating specialists from different specialisms and locations were interviewed and identified benefits and drawbacks of the videoconference meetings (strength)
- The researcher’s presence during video conferenced MDT may have influenced the communication between the centre and the partner, also called ‘Hawthorne effect’ (limitation)
- Only one of the six centres and its preferred partner in the Netherlands was studied (limitation)

Wordcount 3993

For peer review only

For peer review only - http://bmjopen.bmj.com/site/about/guidelines.xhtml

INTRODUCTION

Most tumours in the head or neck region (nasal cavity, paranasal sinuses, lips, mouth, salivary glands, throat or larynx and complex skin malignancies) are fast growing tumours[1]. This implies that a long interval between the moment of referral and the start of the primary treatment (surgery, radiotherapy and/or chemotherapy) can lead to tumour progression with less survival chance[2]. Because of complexity of diagnostic procedures and therapeutic modalities and low volume of patients, head-and-neck cancer care is centralized in multidisciplinary head-and-neck cancer centres[3]. In 1984, the Dutch Head & Neck Society (DHNS) was founded as a scientific organization. Later the DHNS became involved in the nationwide organization of head-and-neck cancer care. As a result, since 1993, head-and-neck cancer patients in the Netherlands are treated in eight head-and-neck cancer centres recognized by the DHNS; six centres have preferred partners[4]. Within each head-and-neck cancer centre, multidisciplinary meetings according to national evidence-based guidelines are mandatory to provide the best diagnostic work up and treatment for patients, and to sustain the quality of care in the oncology centres[5, 6, 7, 8]. Criteria for qualifying as centre: are having the specialisms with expertise to treat the tumour, having the necessary diagnostic and therapeutic facilities and treating at least 200 new patients each year. Partners fulfil the same criteria, but should treat at least 80 new patients.

In 1997, after an informal collaboration period of 4 years, the Medical Centre Leeuwarden became the formal preferred partner of the Head-and-Neck Cancer Centre of the University Medical Centre Groningen[9], further referred to as the "partner" and the "centre". The collaboration of a centre with its partner is based upon trust and sustainable agreements on governance aspects, evidence based multidisciplinary decision-making and use of facilities[10, 11, 12]. The collaboration consists of weekly multidisciplinary team meetings (MDTs) between centre and partner to discuss diagnostic and therapeutic plans. The efficiency of the MDTs is important for decision-making and care pathway management. The centre's MDT regarding diagnostics and treatment involves more than 9 disciplines (details presented elsewhere)[13]. The teams of centre and partner meet face-to-face three times a year, where governance, guidelines and research projects are discussed.

The DHNS and the Dutch Health Care Inspectorate (DHCI) require that all new patients of the partner are discussed in a weekly MDT with the centre[14]. This DHCI requirement can be seen as

For peer review only - http://bmjopen.bmj.com/site/about/guidelines.xhtml

quality control over the partner clinic. Specialists from centre and partner, from the departments of oral and maxillofacial surgery (OMS), ear, nose and throat (ENT) and radiotherapy (RT) participate. This weekly MDT is additional to a local MDT in the hospital where the patient is first seen and will be treated. Initially, these collaborative multidisciplinary weekly meetings were in the centre: three specialists travelled to the oncology centre (2 hours traveling time and 2 hours MDT). When videoconferencing became available, it became the preferred method for this communication.[15, 16] The video-conferenced MDT is scheduled after the local MDT. During the videoconferencing, the partner presents all patient cases, including available imaging, and proposed diagnostic and therapeutic plan. The centre presents complex cases or cases interesting to discuss. Both sides are free to offer recommendations. The team presenting the patient case is responsible for documenting changes when a recommendation is implemented.

Recommendations from both teams to the decision-making regarding diagnostic and therapeutic plans may add value to the quality of patient care[17, 18]. We decided to evaluate the video-conferenced MDT as part of the collaboration agreements because it was time consuming and there was a wish to refocus on benefits and drawbacks.

Research Question

Aim of this study was to analyse the value of video-conferenced MDT in the treatment of head-and-neck cancer patients in the care pathways, resulting in two questions.

1. How often are recommendations given on diagnostic and/or therapeutic plans by the teams during video-conferenced MDT?
2. What benefits and drawbacks of the videoconference are perceived by the specialists in the teams?

DESIGN

This mixed method study[19, 20, 21] had a quantitative part followed by a qualitative part. The primary outcome of the weekly video-conferenced MDT was the percentage of cases in which recommendations on diagnostic and/or treatment plans were given. The secondary outcome were the benefits or drawbacks of the MDT video conference perceived / experienced by the participating

For peer review only - http://bmjopen.bmj.com/site/about/guidelines.xhtml

specialists. In the study period, the teams acted conform the DHCI requirement that all patients of the partner should be presented in a multidisciplinary meeting with the centre.

Videoconferencing equipment used

The video-conferenced MDT was held in dedicated multidisciplinary meeting rooms, where screens can be operated with two to four computers with monitors. While the patient data is presented on the first screen, teams can see each other on the second screen. The videoconferencing is operated via the 'Webex'-application and a camera. Both locations call into a special safe 'chat room'.

Centre: dedicated 20-seat VC room with three screens - beamers (software / provider Kinly; bandwidth 2 Mbps) and five camera inputs. Four computer stations, one dedicated for Radiology showing PACS Imaging.

Partner: dedicated 10-seat VC room with one screen with possibility to see patient data and the other team; one computer log-on to patient dossiers showing data and imaging.

Patient data

Data of all patients presented by one of the teams during the video-conferenced MDT videoconferences between September 2016 and February 2017 were included. The tumour localization, histology and tumour stage were registered for all patients that were presented.

Patient involvement in study design

Patients were not involved in the study because the main purpose of the study was to evaluate the added value of the DHCI requirement in a weekly video-conferenced MDT.

Quantitative part

Sample size calculation recommendations

In a 4-week pilot study of 4 sessions including 46 cases, carried out 9 months before study start, we found that in approximately 20% of cases a recommendation was given. To estimate this percentage with a 10% precision (95 % confidence interval: 15.5 % to 25.4 %) would require 250 cases. On average, 15 cases were discussed at each weekly video-conferenced MDT. We estimated that six months would be sufficient to acquire the necessary 250 cases. The pilot study was also used to operationalize the primary outcome measure.

Recommendation registration

For peer review only - http://bmjopen.bmj.com/site/about/guidelines.xhtml

165 Recommendations were registered with the relevant data from electronic and written medical records on a clinical registration form by LvH during the videoconference. Each recommendation was assessed by the two teams with respect to change impact (minor or major, Table 1a) on the diagnostic and/or therapeutic plan, case complexity, use of national multidisciplinary guidelines for the diagnostic and/or treatment plan, and comorbidity of the patient (Table 1b). LvH registered the given recommendation with the relevant data; JdV and JR verified the registrations. During the videoconferencing sessions, field notes were taken by LvH.

Table 1. Definitions of change impact and case complexity

Table 1a Operational definitions of major and minor changes in diagnostic or treatment plan

	diagnostic plan	treatment plan	remarks
minor	additional more-detailed MRI or CT-thorax of the area already imaged	logistic change	
major	additional MRI or CT-thorax in a different area from the area already imaged	change in modality: adding or deleting a therapeutic modality; surgery radiotherapy or chemotherapy	
criterion	addition of diagnostic plan in a different area than already investigated	adding or deleting a treatment modality from the treatment plan in the proposed or in a different area	after the major/minor decision is made, the decision registered in the research form will be verified by both specialists (giver and receiver)

Table 1b Operational definition of case complexity

	modality	guideline	comorbidity
not complex	unimodal treatment	diagnosis and treatment follows guideline	no comorbidity
complex	multimodal treatment	diagnosis and/or treatment does not follow guideline	comorbidity
remark	- unimodal:<br>surgical procedure<br>chemotherapy<br>primary radiotherapy<br>- multimodal:<br>reconstruction surgery<br>chemo- or bio-radiotherapy	which guidelines are followed	

Statistical analysis

For peer review only - http://bmjopen.bmj.com/site/about/guidelines.xhtml

Differences in age, gender, tumour localization and tumour histology (ICD(O))[22], and tumour stage between cases presented by the centre and those presented by the partner were analysed using t-test for independent samples, Chi-Squared test, and Chi- Squared test exact procedure if requirements for the Chi-Square test were not met. Statistical analyses were performed using SPSS 23.0 for Windows software. In all analyses, statistical significance was set at the 5% level.

Qualitative part

Interviews

Semi-structured interviews were conducted with six medical specialists that attended the meetings most frequently, one from the OMS-, ENT- and RT-department of each team, to explore the added value of the video-conferenced MDT. The field notes taken by the researcher during the video-conferenced MDT were used to develop the questions for the semi-structured interviews. After receiving verbal informed consent from the specialists, the semi-structured interviews started with providing information about the recommendations given. Thereafter it continued with the open question 'What do you think is the value of the videoconference between the head-and-neck cancer centre and their preferred partner?'. LvH then guided the interview using a short topic list including 'added value' and 'perceived possibilities for change or improvement in the video-conferenced MDT' (Textbox 1). The different topics were introduced in a flexible way, and the interviews took the form of natural conversations.

For peer review only

Textbox 1 Interview Guide

Topics	Questions
Added value videoconferencing	What do you think is the added value of the video-conferenced MDT between the head-and-neck cancer centre (centre) and their preferred partner (partner)?<br>Could you mention strong points of the video-conferenced MDT?<br>Could you give examples?<br>Could you name points for improvement?<br>Could you mention examples?
Role of specialism in videoconference	What do you think the role of a specialist is in the video-conferenced MDT between centre and partner?<br>The consultation is required by the Dutch Head and Neck Society and the Dutch Health Care Inspectorate, how usefulness do you think it is?<br>Would you advise stopping the consultation if it was not mandatory?
Results interpretation	Have you given recommendations to the centre/partner?<br>Have you received recommendations from the centre/partner?<br>Could you indicate what the difference is between peer consultation and giving a recommendation?<br>What do you think would be an ideal video-conferenced MDT? Could you

For peer review only - http://bmjopen.bmj.com/site/about/guidelines.xhtml

explain your answer?
What do you think could be adjusted in the video-conferenced MDT to make the consultation more effective and more efficient?

Interviews lasted between 25 and 40 minutes, were audio recorded and transcripts of the interviews were made. The participants were asked to review the transcripts and extracted quotes, related to perceived added value, possible improvements and the role of a specialist in the video-conferenced MDT.

Thematic analysis

Quotes were anonymized and coded for their relevance to possible benefits or drawbacks for the collaboration between the teams and for patient care. The first stage of this inductive analysis of the interviews involved two authors, JR and JdV, in an initial open coding procedure that resulted in a list of codes corresponding closely to the text fragments extracted from the six interviews. The codes were placed in a coding tree using a thematic analysis approach with main themes recommendations, added value, collaboration and planning[23, 24]. Codes were judged as being a benefit or a drawback. Any disagreements during the coding were discussed between the coders and the researcher[25]. After the preliminary results were collated, for credibility a member check was performed with participants.[26] The Clinical Research Office performed a planned quality check on data management.

RESULTS

Quantitative analysis

From September 2016 to February 2017, 82 patients were presented by the centre and 177 by the partner in 18 weekly video-conferenced MDTs (Table 2). In this period of 22 weeks, three meetings were cancelled due to a 'medical complication meeting', a technical problem to connect and a holiday recess. Further, the researcher could not attend one session.

For peer review only

For peer review only - http://bmjopen.bmj.com/site/about/guidelines.xhtml

Table 2 Patients and their tumour characteristics, as presented during videoconference meetings

Number of patients (total n=259)	*centre (n=82)*		*partner (n=177)*		statistics, p =
(n=number of available data)	*mean*	*SD*	*mean*	*SD*	
**Age** (Mean, SD)	67.8	15.2	66.7	16.1	*(t-test) .533*
**Gender (n=259)**	*n*	*%*	*n*	*%*	*(Chi²) .394*
Female	27	10	68	26	
**Tumour localization (n=206*)**	*n*	*%*	*n*	*%*	*(Chi²-exact) < .001*
Lip (C00)	3	3	4	2	
Oral cavity	21	23	29	12	
Tongue (C01, C02)	6	-	11	-	
Gums (C03)	5	-	7	-	
Floor of mouth (C04)	4	-	4	-	
Oral cavity, unspecified (C05, C06, C14)	6	-	7	-	
Major salivary glands (C07, C08)	2	2	7	3	
Oropharynx (C09,C10)	7	8	6	2	
Nasopharynx (C11)	0	0	0	0	
Nasal Cavity (C30)	2	2	3	1	
Hypopharynx (C12, C13)	5	5	5	2	
Sinus (C31)	3	3	3	1	
Larynx (C32)	10	11	15	6	
Bronchus and Lung (C34)	0	0	5	2	
Hematologic and reticuloendothelial systems (C42)	0	0	11	5	
Skin (C44)	14	15	35	14	
Lymph nodes (C77)	2	2	1	0	
Unknown (C80)	3	3	0	0	
Miscellaneous (C20, 33, 41, 49, 50, 64, 73)	3	3	7	3	
Unknown (C80)	3	3	0	0	
**Morphology or cell type (n=259)**	*n*	*%*	*n*	*%*	*(Chi²) < .001*
Squamous cell carcinoma (SCC)	57	72	78	44	
Basic cell carcinoma (BCC)	3	4	6	3	
Melanoma	0	0	11	6	
Miscellaneous malignant	7	9	9	5	
Benign	2	2	18	10	
Infection – premalignant abnormalities	2	2	12	7	
Miscellaneous	11	13	43	24	
**T-stage (n=159**)**	*n*	*%*	*n*	*%*	*(Chi²) < .001*
T1	13	14	42	17	
T2	20	22	20	8	
T3	8	9	9	4	
T4	25	27	14	6	
Tx	7	8	1	1	

*= only tumour localization if tumour diagnosed; **= only TNM-code if firstly diagnosed, so there are more patients in which 'localization' is known (i.e. for relapse or tumour residue or metastases).
In total 336 cases presented: 93 by centre and 243 by partner.

For peer review only

For peer review only - http://bmjopen.bmj.com/site/about/guidelines.xhtml

Most of the centre's patients (71 out of 82 – 86%) were presented only once, nine were presented twice (11 %), one patient was discussed three times and another four times. Whereas 111 patients were presented only once (63%) by the partner. Generally patients of the partner where presented twice or three times: the first time their diagnostic plan, the second time the therapeutic plan and sometimes surgical results the third time (55 out of 177 – 31%). Only one patient was discussed four times; five patients on the partner's list were not discussed at the first opportunity because imaging was not complete.

The partner presented significantly ($p < .001$) more cases with infections that were initially suspected malignancy, T1-stage patients and non-complex cases. Tumour localization and histology differed also significantly between centre and partner (Table 2). In 61% of the 18 videoconferences both teams were complete; the centre team was not complete in 22% (n=4) and, in 17% (n=3), the partner team was not complete. On those occasions one of the other specialisms would present the cases, for example OMS for ENT. The centre's ENT department was represented in most meetings by an ENT-specialist training to be a head-and-neck oncology surgeon. The centre presented on average 5.2 (SD 2.4) cases per videoconference, the partner presented on average 13.5 (SD 3.9) cases.

Recommendations given

Recommendations were given in eight of the 336 cases presented (2%; 95% confidence interval: 1 to 5%) relating to eight of the 259 patients (3%; 95% confidence interval: 1 to 6%).

Of these recommendations, five were major and three minor (Table 3). Four recommendations concerned diagnostic plans, and four treatment plans. On three of the eight occasions when a recommendation was given, the centre's team was incomplete with one of the three specialisms absent. Seven of the eight recommendations were given by OMS specialists, and five of the eight were related to ENT patients. Seven of the eight instances occurred on a patient's first presentation and the other one during a second presentation although, in this case, the imaging had not been complete the first time. In general, recommendations were given related to the more complex cases, but not necessarily patients with comorbidity or those with more advanced tumours. About 70% of case were 'formalities' or 'routine patients', meaning patients that fitting the guidelines (well-defined tumours with limited regional metastases and without comorbidity).

For peer review only - http://bmjopen.bmj.com/site/about/guidelines.xhtml

Table 3 Recommendation and its specifics

No	Recommen-dation	Who	To whom	Team complete?	Recommen-dation (short)	Change impact, diagnosis or treatment phase	Patient status (ICD-code, TNM-classification, histology; case complexity, guideline used and comorbidity)					
							ICD	TNM	histology	com-plex?	guide-line?	comor-bid?
1	2016G10-1 28-09-2016	OMS partner	ENT centre	yes	give patient choice of expectative treatment	major, treatment	C44	T2N0M0	SCC	yes	no	yes
2	2016L14-1 28-09-2016	OMS centre	OMS partner	yes	ultrasound guided biopsy	minor, diagnosis	-	-	maligne lympho-ma	no	yes	no
3	2016G32-1 26-10-2016	OMS partner	OMS centre	centre not	use methotrexate to identify malignancy	minor, treatment	C00	T1N0M0	SCC	yes	yes	no
4	2016G39-1 23-11-2016	OMS partner	ENT centre	yes	change surgery approach to retain functionality	major, treatment	C00	T2N0M0	adenoid cystic carcinoma	yes	no	no
5	2016G40-1 23-11-2016	OMS partner	ENT centre	yes	try PDT	major, treatment	C01	T4aN0M0	SCC	yes	no	no
6	2016G51-1 14-12-2016	OMS partner	ENT centre	centre not	consult Ophthalmo-logy	major, diagnosis	C44	T2N0M0	BCC eye corner	yes	no	yes
7	2016L90-2 14-12-2016	OMS centre	ENT partner	centre not	new biopsy	major, diagnosis	C31	T3NxM0	Melan.	yes	yes	yes
8	2017L123-1 04-01-2017	RT centre	OMS partner	yes	add MRI	minor, diagnosis	C07	T1N0M0	SCC	yes	yes	no
Squamous cell carcinoma (SCC), BCC = Basal cell carcinoma, Melan. = Melanoma; MRI = Magnetic Resonance Imaging; PDT = Photo Dynamic Therapy												

For peer review only

Qualitative analysis – specialist interviews

During May 2017 six interviews were held. From the transcripts of the six interviews, 107 quotes were registered. During the coding procedure, items were placed in a coding tree with relevance to the primary research question (recommendations given) and the secondary research question (perceived benefits and drawbacks) by the researcher in consultation with the coders. For each major theme, minor themes were derived from the researcher's field notes. In total 282 scores were given (Table 4). In several instances the quotes were scored differently although the inter-coder agreement was acceptable given the possible 37 codes to choose from.

Benefits were more frequently mentioned by specialists of the partner, and the drawbacks more frequently by specialists of the centre. But the majority of codes had a positive connotation for the video-conferenced MDT (Table 4).

For peer review only - http://bmjopen.bmj.com/site/about/guidelines.xhtml

Table 4 Coding tree evaluation video-conferenced MDT

Coding tree			Pos?	Code	Code description	Part-ner	Centre	Total
Videoconferencing	Recom-menda-tion	Nuance	+	22	video-conferenced MDT is mostly ‘inter-collegial consultation’	3	3	6
			+	14	recommendations are nuances, not a totally different medical procedure or diagnostic/treatment plan for a specific patient	7	10	17
		Follow-up traceable?	+	6	suggestions are taken from others	1	2	3
			+	20	there is no patient-level evaluation on the implementation of medical procedures agreed, question of trust	3	2	5
			-	34	sometimes decisions are already taken in relation to continuity of treatment	1	1	2
		Aligning	+	1	fine-tuning or aligning medical procedures	10	10	20
			+	9	continue routine cases discussion to prevent deviation from medical procedures	2	2	4
		Knowledge	0	32	besides videoconferencing also bilateral consultation via telephone	4	1	5
			+	37	keep ‘know how’ with routine cases	1	2	3
	Added Value?	Video-confe-renced MDT	+	8	added value for complex cases vs. routine cases	21	24	45
			-	15	little added value	8	1	9
			0	27	discuss radio-therapeutic scheme	2	2	4
			-	29	non-complex cases or ‘formalities’ are communicated because it is mandatory, no added value	7	1	8
			+	30	recommendation given to own discipline	5	1	6
		Team completeness	+	4	presence of all three disciplines is essential	3	4	7
			+	11	expertise (good) of physician is important	5	3	8
			0	23	add presence of medical oncology discipline as expertise	2	2	4
	Collabo-ration	Communi-cation	0	2	working together requires communication	8	2	10
			+	10	at both locations working methods are comparable through video-conferenced MDT	5	2	7
			-	19	initially it was good to consult, added value decreased because teams have grown towards each other	1	1	2
		Trust	+	5	respectful collaboration	3	7	10
			+	7	mutual trust	4	5	9
			+	13	important to know the partner, not only via videoconferencing; good for cohesion	8	7	15
		Expertise	-	18	centre member does not think videoconferencing necessary, because partner should be trusted as such	2	4	6
			+	26	expertise and new developments from centre to partner	2	2	4
		DHCI	0	21	video-conferenced MDT between centre and partner is a national agreement or policy	2	3	5

For peer review only

For peer review only - http://bmjopen.bmj.com/site/about/guidelines.xhtml

Coding tree			Pos?	Code	Code description	Part-ner	Centre	Total
		requirement	-	31	the national policy – to discuss all cases including routine cases – between centre and partner is perceived as out-dated	7	2	9
	Plan-ning	Logistics	-	16	stressful, considering other videoconferences	3	6	9
			0	17	integrate video-conferenced MDT in the hospital's MDT for centre and partner	5	7	12
		Preparation	-	12	improve format of patient presentation	1	1	2
			+	24	good preparation is important	5	4	9
		Commitments	+	25	starting and stopping the video-conferenced MDT on time is important	4	1	5
			0	33	possibly cancel video-conferenced MDT when nothing to discuss	1	1	2
		Equipment	+	3	technique always flawless	1	1	2
			-	35	sometimes video-conferenced MDT did not take place due to technical malfunction	1	1	2
			-	36	placement of monitor in the room hinders colleagues and hampers interaction	2	2	4
Scientific Research			0	28	bias through research setting because researcher is present as observer (Hawthorne effect)	1	1	2
					Total quotes	151	131	282

This coding tree has major and minor themes that were derived from the primary research question (recommendations given), the secondary research question (added value as described in benefits and drawbacks perceived) and minor themes derived from researcher's field notes. One code was related to the research situation.

'Pos?' refers to the question: has this code a positive connotation or benefit? + = yes, 185 scores; 0 = neither positive nor negative, 42 scores; - = no, 55 scores.

The amount of codes given is given for the partner, the centre and in total. DHCI is short for Dutch Health Care Inspectorate.

For peer review only

Six main items were important according to the specialists (quotes in italic).

1. The videoconference adds value when discussing complex cases, through assisting in fine tuning and aligning medical procedures (code 1, 20x);

A patient is presented about which the own team had some discussion, that can be discussed with the partner. In that manner, you get a confirmation or advice to change your treatment plan. This advice can be given by the same specialism, but also by other members of the head-and-neck oncology team (ENT).

2. Communication is essential for cooperation between teams (code 2, 10x), furthermore it is important to know the partner well, not only via videoconferencing (code 13, 15x), and to interact respectfully (code 5, 10x) with mutual trust (code 7, 9x).

The most important feature of the video-conferenced MDT is to communicate with each other on substantive medical matters, to be on speaking terms, and to know each other (RT).

During the videoconferencing, we respect each other, we listen to each other and we are open to each other's additional comments. We trust each other as partners (OMS).

3. Recommendations are suggested alternatives on diagnostic modalities and treatment plans for specific patients (code 14, 17x).

The video-conferenced MDT has the character of a collegial discussion, in which in collaboration the best diagnostic or treatment plan for your patient is reached. Confirmation on your treatment plan adds value too (OMS).

4. For routine cases that fall within guideline for treatment, the videoconference meeting adds little value as for changes in medical content, it can even irritate the participants in such cases (code 15, 9x).

The video-conferenced MDT sometimes changes the treatment plan for an individual patient. The videoconference is not the meeting where new procedures or guidelines are developed (RT).

5. There is a wish to integrate the videoconference with the site multidisciplinary meeting in both hospitals, the centre and the partner (code 17, 12x).

Integration of the two local multidisciplinary meetings with the video-conferenced MDT could be valuable (ENT).

For peer review only - http://bmjopen.bmj.com/site/about/guidelines.xhtml

6. The DHCI requirement (discuss all the partner's cases) has no added value. It is seen as old-fashioned or out-dated (code 29, 8x).

It is better to prepare at a high level and discuss, than to present all the patients and deal with each one briefly. Mutual preparation on special request could have added value, for example a literature search on a complex osteosarcoma case (OMS).

DISCUSSION

Our results show that the added value of the weekly video-conferenced MDT between the head-and-neck cancer centre and the partner hospital was small given the few recommendations made on the initial diagnostic and/or treatment plan. Nevertheless, the specialists from both sites recognized the importance of keeping their medical viewpoints aligned through this type of communication. Whenever discussing complex cases in which a major change was recommended (in 5 of the 8 recommendations), for example to change the surgical approach to save functionality of organs or tissue, the recommended change in treatment had a large impact for that patient (Table 3).

The data from the interviews suggest that especially complex patients would benefit from inter collegial consultation via video-conferenced MDT. If the teams were not obliged to discuss so many routine cases, they could use the time saved to prepare and discuss complex cases in greater depth[27]. The specialists said that they did not want to stop the video-conferenced MDT, because they appreciate reflecting on diagnostic and treatment plans with trusted expert colleagues.

Because of an increase in patients to be presented in the meeting, we were looking for a more efficient meeting, which could be reached not discussing the 'formalities' or 'routine patients' (about 70% of patients); developing an evidence based working method would need more research. This result is supported by a large survey in the UK after 10 years of use of an MDT format, where specialists also said they wanted to change many components and refocus to spend more time on complex cases in detail[18].

The qualitative part of this study showed that medical specialists perceived added value in discussing complex cases in a collegiate consultation, because the other team offers a fresh perspective by hearing the case 'as new'. Although remarks were often about nuances, the confirmation on the

For peer review only - http://bmjopen.bmj.com/site/about/guidelines.xhtml

chosen treatment by the other team was experienced as helpful. This view is supported in literature where medical specialists found videoconferencing useful in at least one aspect of their practice[10].

An important requirement to communicate through videoconference is that participants know each other from personal meetings, to support mutual trust and respect as the basis for cooperation. The finding that collaboration and cooperation improves when each discipline understands each other's roles and that specialties working together for a long time do not need many words to come to a decision was supported previously[17, 28].

The video-conferenced MDT can be used to introduce and discuss new developments, protocols and guidelines leading to comparable quality of care in both locations. Comprehensive cancer centre teams working together over videoconferencing with a peripheral hospital team, reviewing radiotherapy planning align their treatment plans (7% major and 21% minor changes)[16] and speed up follow-up appointments[15].

The video-conferenced MDT differs from the local MDT: complex cases are discussed with a second 'expert team' of head-and-neck oncology specialists. The patients treated by the centre and partner are similar, although diagnostics and treatment might differ slightly[29], only in case of rare tumours that need skull base surgery patients travel from partner to centre. In our study the significant differences in tumour localization, cell type and tumour stage between sites are a consequence of 'the DHCI requirement' whereas the 'centre' could decide which of its patients would make an interesting case for discussion. Consequently, the partner presents 3 to 4 times as many patients as the centre. One third of these (31%) reappeared in the subsequent videoconferences, checking extra diagnostic information, treatment plan and need for adjuvant therapy. Most of these presentations were seen as a 'formality'.

The perceived value of the video-conferenced MDT might be influenced by the expertise of specialists. The recommendations given during the evaluation period were mostly given to ENT by an OMS oncologist who had considerably more clinical experience than his opposing colleague had, and was one of the instigators of the collaboration. It could be that recommendations given were accepted more easily if given by a more experienced specialist[12]. Videoconferencing enables specialists

For peer review only - http://bmjopen.bmj.com/site/about/guidelines.xhtml

acquiring experience with presenting complex oncology patients and with decision-making in teams[6, 17].

Limitations of this study

Contrary to our findings from the 4-week pilot study (n=46), where advice was offered in 20% of the presented cases, the actual 2% recommendations is much lower. Although it is difficult to explain this difference in amount of 'agreed recommendations', we think that the pilot served mainly as a feasibility check, that helped us to define our research questions and to operationalize the definitions. Other factors may also have played a role in the difference between the pilot and the actual study. Firstly, the long-lasting collaboration between the centre and the partner had led to a high level of alignment on diagnostic and therapeutic 'strategies' or medical viewpoints. Secondly, the participants were not blinded for the research question. Thus, awareness of being part of an experiment may have led to a drive to perform well and to present the patients with an optimal diagnostic and treatment plan (Hawthorne effect). Additionally presence of the researcher might have influenced the communication between centre and partner. Often the teams mentioned that the other team was asked to give collegial advice and therefore a suggestion was not always seen as a recommendation. This nuance could also be interpreted as a social desirable answer, possibly due to the long existing collaboration between the centre and the partner before study start. Thirdly, some patient cases were only presented as interesting to discuss. Finally, during the pilot study the advice given was not assessed for its impact.

In this study, we evaluated the added value of a video-conferenced MDT between one oncology centre and its preferred partner. In line with other studies[30, 31], this study showed that, in addition to a quantitative result (number of recommendations), it is important to reflect on the situation through an interview process (qualitative results) before starting to implement improvements. The interviews showed that specialists from both centre and partner support the idea of sustainable collaboration, but they do not support the view implicit in the DHCI requirement that the centre should act as means of quality control for the partner[32]. Our findings on video-conferenced MDTs find support elsewhere in terms of the positive results on teams working together[33-35]. Other studies have shown that more research is needed to understand the effects of video-conferenced MDT on patient outcomes, such as

For peer review only - http://bmjopen.bmj.com/site/about/guidelines.xhtml

For peer review only

finance including resource usage[36, 37], what fields of specialisms could benefit from the medium[28, 38], participant satisfaction[39], throughput times[40] and self-management for patients[41].

In summary we believe that the DHCI requirement (the partner should discuss all patients with the centre) is unnecessary in the case of routine patients, since it does not add value to the quality of their treatment. It is more useful to spend time to discuss complex cases in greater detail. We propose the following measures that will add value to the weekly video-conferenced MDT:

1. All the participating medical specialists should be granted freedom to select only complex or interesting cases that could serve to keep medical procedures aligned.
2. The partner should not be obliged to present cases seen as ‘routine patients’ since this does not add value.
3. The video-conferenced MDT should be organized as an integral part of the partners’ MDT and not as a separate weekly meeting.
4. Accepted, mature processes should be regularly reassessed and refocused in order to enable new collaboration strategies.

Based on our findings on the added value of the multidisciplinary videoconference between the head-and-neck centre and its partner and our suggestions for improvements, we would advise the DHNS, along with healthcare policymakers, to reconsider the DHCI requirement.

In our study, we found that there are clinical and practical implications on how and when to start with videoconferencing instead of meetings with physical attendance. Videoconferencing must be seen as a supportive medium for communication within a sustainable collaboration of parties that understand each other’s roles and work with guidelines or protocols.

Participants of a videoconference should:

1. Know each other, and meet face-to-face on a regular basis, which serves cohesion (management meetings on governance, guideline developments and research projects are ideal for this purpose).
2. Respect each other as ‘expert / knowing’ colleague and know each other’s role in the multidisciplinary treatment of patients.
3. Trust each other in follow-up of changes to diagnostic and treatment plans.

For peer review only - http://bmjopen.bmj.com/site/about/guidelines.xhtml

For peer review only

In view of the above mentioned implications we would not recommend starting with videoconferencing for multidisciplinary meetings if a majority of participants do not know each other.

CONCLUSIONS

The video conferenced MDT has added value in the collaboration and in the care pathway management. When interpreting national multidisciplinary guidelines, centre and partner align their medical policies. This leads to a more efficient use of resources and work force.

Conversely, discussing non-complex cases is seen as a burden, and the DHCI requirement to discuss all the partners' cases as out-dated.

LIST OF ABBREVIATIONS

DMD	Doctor of Dental Medicine
DDS	Doctor of Dental Surgery
MDT	Multidisciplinary Team meeting
DHCI	Dutch Health Care Inspectorate
DHNS	Dutch Head & Neck Society
ENT	Ear, Nose and Throat
ICD(O)	International Classification of Diseases (of Oncology)
MeSH	Medical Subject Headings
OMS	Oral and Maxillofacial Surgery
RT	Radiotherapy
SPSS	Statistical Package for Social Sciences
UMCG	University Medical Center Groningen

DECLARATIONS

Ethics approval and consent to participate

This prospective observational study on decision-making analysis was checked by the Medical Ethics Review Board of the UMCG (2016, ref. M16.194909), the Netherlands. They concluded that the study is not a 'clinical research study with human subjects' as meant in the Medical Research Involving Human Subject Act (WMO). Informed consent was not required. The Dutch law requires also a privacy statement from the partner in the study, the Medical Centre Leeuwarden (2016, nWMO 187).

For peer review only

For peer review only - http://bmjopen.bmj.com/site/about/guidelines.xhtml

Data sharing statement

Datasets will be available from the corresponding author on request.

Consent for publication

Not applicable

Competing interests: No authors have competing interests.

Funding: This research received no specific grant from any funding agency in the public, commercial or not-for-profit sectors.

Authors' Contribution

LvH was involved in the study design and concept; carried out the study; performed the statistical analysis and the analysis and interpretation of the data; and drafted the manuscript. PD, KA, JdV and JR, the supervisor, were involved in the study design and concept, analysis and interpretation of the data, and revision of the manuscript. JdV and JR were involved in the coding of the interview quotations, together with LvH. GH, JvdH, KvdL and OW were involved in the acquisition of the data and the revision of the manuscript.

All authors read and approved the final manuscript.

Acknowledgements

This research was sponsored by the University Medical Centre Groningen.

Authors' information

The University Medical Center Groningen is developing patient centred care pathways for diverse patient groups including laws and regulations for quality and patient safety. LvH, JR are working in cooperation with KA to research care pathway implementation in the Comprehensive Cancer Center Groningen and to develop quality and safety indicators, i.e. process indicators that predict performance of care pathways and sustainable patient outcome.

REFERENCES

1 Waaijer A, Terhaard CH, Dehnad H, et al. Waiting times for radiotherapy: consequences of volume increase for the TCP in oropharyngeal carcinoma. *Radiother Oncol* 2003;66:271-6.

2 Ouwens M, Hermens R, Hulscher M, et al. Development of indicators for patient-centred cancer care. *Support Care Cancer* 2010;18:121-30 doi:10.1007/s00520-009-0638-y; 10.1007/s00520-009-0638-y.

For peer review only - http://bmjopen.bmj.com/site/about/guidelines.xhtml

3 Policy head and neck cancer care 2013 (in Dutch). Available at: http://www.nwhht.nl/organisatie/missie.

4 Halmos GB, Bras L, Siesling S, et al. Age-specific incidence and treatment patterns of head and neck cancer in the Netherlands-A cohort study. *Clin Otolaryngol* 2018;43:317-24 doi:10.1111/coa.12991 [doi].

5 Fleissig A, Jenkins V, Catt S, et al. Multidisciplinary teams in cancer care: are they effective in the UK?. *Lancet Oncol* 2006;7:935-43 doi:S1470-2045(06)70940-8 [pii].

6 Ruhstaller T, Roe H, Thurlimann B, et al. The multidisciplinary meeting: An indispensable aid to communication between different specialities. *Eur J Cancer* 2006;42:2459-62 doi:S0959-8049(06)00555-7 [pii].

7 Ouwens MM, Hermens RR, Hulscher MM, et al. Impact of an integrated care program for patients with head and neck cancer on the quality of care. *Head Neck* 2009;31:902-10 doi:10.1002/hed.21041; 10.1002/hed.21041.

8 Dutch National Cancer Control Programme. Progress Report on Cancer Control in the Netherlands, 2005-2010 (Dutch NCCP, Nationaal programma kankerbestrijding). 2010.

9 Cijfers over kanker / Figures on cancer. Available at: http://www.cijfersoverkanker.nl. Accessed May, 6, 2017.

10 Norum J, Jordhoy MS. A university oncology department and a remote palliative care unit linked together by email and videoconferencing. *J Telemed Telecare* 2006;12:92-6 doi:10.1258/135763306776084374 [doi].

11 Bydder S, Nowak A, Marion K, et al. The impact of case discussion at a multidisciplinary team meeting on the treatment and survival of patients with inoperable non-small cell lung cancer. *Intern Med J* 2009;39:838-41 doi:10.1111/j.1445-5994.2009.02019.x [doi].

12 Slavova-Azmanova NS, Johnson CE, Platell C, et al. Peer review of cancer multidisciplinary teams: is it acceptable in Australia?. *Med J Aust* 2015;202:144-7 doi:10.5694/mja14.00768 [pii].

13 van Huizen LS, Dijkstra PU, van der Laan BFAM, et al. Multidisciplinary first-day consultation accelerates diagnostic procedures and throughput times of patients in a head-and-neck cancer care pathway, a mixed method study. *BMC Health Serv Res* 2018;18:820,018-3637-1 doi:10.1186/s12913-018-3637-1 [doi].

14 SONCOS, Dutch policy on multidisciplinary standardization for oncological care (in Dutch): Soncos Normeringsrapport 7, pages 22-26. Available at: https://www.soncos.org/wp-content/uploads/2019/02/Soncos_norm-rapp2019-v7.pdf. Accessed 07/11, 2019.

15 Farris G, Sircar M, Bortinger J, et al. Extension for Community Healthcare Outcomes-Care Transitions: Enhancing Geriatric Care Transitions Through a Multidisciplinary Videoconference. *J Am Geriatr Soc* 2017;65:598-602 doi:10.1111/jgs.14690 [doi].

16 Seeber A, Mitterer M, Gunsilius E, et al. Feasibility of a multdisciplinary lung cancer videoconference between a peripheral hospital and a comprehensive cancer centre. *Oncology* 2013;84:186-90 doi:10.1159/000345314 [doi].

For peer review only

17 Gagliardi A, Smith A, Goel V, et al. Feasibility study of multidisciplinary oncology rounds by videoconference for surgeons in remote locales. *BMC Med Inform Decis Mak* 2003;3:7,6947-3-7. Epub 2003 Jun 19 doi:10.1186/1472-6947-3-7 [doi].

18 Meeting patients' needs. Improving the effectiveness of multidisciplinary teams meetings in cancer services. Available at: https://www.cancerresearchuk.org/sites/default/files/full_report_meeting_patients_needs_improving_the_effectiveness_of_multidisciplinary_team_meetings_.pdf. Accessed 07/11, 2019.

19 Charmaz K. Contructing Grounded Theory. A Practical Guide through Qualitative Analysis. London: Sage Publications 2006.

20 Plochg T, Juttman RE, Klazinga NS, et al. Handbook health research (Handboek gezondheidszorgonderzoek). Houten: Bohn Stafleu van Loghum 2007.

21 O'Brien BC, Harris IB, Beckman TJ, et al. Standards for reporting qualitative research: a synthesis of recommendations. *Acad Med* 2014;89:1245-51 doi:10.1097/ACM.0000000000000388 [doi].

22 Vlaams Kankerregistratienetwerk. International Classification of Diseases for Oncology, third edition, updates (in Dutch): Vlaams Kankerregistratienetwerk 2011:240.

23 Creswell J, Plano Clark V. Designing and conducting mixed methods research. Los Angeles: Sage 2011.

24 Tong A, Sainsbury P, Craig J. Consolidated criteria for reporting qualitative research (COREQ): a 32-item checklist for interviews and focus groups. *Int J Qual Health Care* 2007;19:349-57 doi:mzm042 [pii].

25 Gioia DA, Gorley KG, Hamilton AL. Seeking Quality Rigor in Inductive Research: Notes on the Gioia Methodology. *Organizational Research Methods* 2012;16:15,16-31 doi:10.1177/1094428112452151.

26 Birt L, Scott S, Cavers D, et al. Member Checking: A Tool to Enhance Trustworthiness or Merely a Nod to Validation?. *Qual Health Res* 2016 doi:1049732316654870 [pii].

27 Xylinas E, Roupret M, Kluth L, et al. Collaborative research networks as a platform for virtual multidisciplinary, international approach to managing difficult clinical cases: an example from the Upper Tract Urothelial Carcinoma Collaboration. *Eur Urol* 2012;62:943-5 doi:10.1016/j.eururo.2012.08.022 [doi].

28 Stevens G, Loh J, Kolbe J, et al. Comparison of recommendations for radiotherapy from two contemporaneous thoracic multidisciplinary meeting formats: co-located and video conference. *Intern Med J* 2012;42:1213-8 doi:10.1111/j.1445-5994.2012.02817.x [doi].

29 de Ridder M, Balm AJM, Baatenburg de Jong RJ, et al. Variation in head and neck cancer care in the Netherlands: A retrospective cohort evaluation of incidence, treatment and outcome. *Eur J Surg Oncol* 2017;43:1494-502 doi:S0748-7983(17)30364-5 [pii].

30 Helck A, Matzko M, Trumm CG, et al. Interdisciplinary expert consultation via a teleradiology platform--influence on therapeutic decision-making and patient referral rates to an academic tertiary care center. *Rofo* 2009;181:1180-4 doi:10.1055/s-0028-1109733 [doi].

For peer review only

For peer review only - http://bmjopen.bmj.com/site/about/guidelines.xhtml

31 Ottevanger N, Hilbink M, Weenk M, et al. Oncologic multidisciplinary team meetings: evaluation of quality criteria. *J Eval Clin Pract* 2013;19:1035-43 doi:10.1111/jep.12022 [doi].

32 Stoffels AR. Cooperation among medical specialists: pain or gain? 2008.

33 Axford AT, Askill C, Jones AJ. Virtual multidisciplinary teams for cancer care. *J Telemed Telecare* 2002;8 Suppl 2:3-4 doi:10.1177/1357633X020080S202 [doi].

34 Barry N, Campbell P, Reed N, et al. Implementation of videoconferencing to support a managed clinical network in Scotland: lessons learned during the first 18 months. *J Telemed Telecare* 2003;9 Suppl 2:S7-9 doi:10.1258/135763303322596110 [doi].

35 Qaddoumi I, Mansour A, Musharbash A, et al. Impact of telemedicine on pediatric neuro-oncology in a developing country: the Jordanian-Canadian experience. *Pediatr Blood Cancer* 2007;48:39-43 doi:10.1002/pbc.21085 [doi].

36 Sezeur A, Degramont A, Touboul E, et al. Teleconsultation before chemotherapy for recently operated on patients. *Am J Surg* 2001;182:49-51 doi:S0002-9610(01)00662-6 [pii].

37 Kunkler IH, Fielding RG, Brebner J, et al. A comprehensive approach for evaluating telemedicine-delivered multidisciplinary breast cancer meetings in southern Scotland. *J Telemed Telecare* 2005;11 Suppl 1:71-3 doi:10.1258/1357633054461804 [doi].

38 Murad MF, Ali Q, Nawaz T, et al. Teleoncology: improving patient outcome through coordinated care. *Telemed J E Health* 2014;20:381-4 doi:10.1089/tmj.2013.0100 [doi].

39 Careau E, Dussault J, Vincent C. Development of interprofessional care plans for spinal cord injury clients through videoconferencing. *J Interprof Care* 2010;24:115-8 doi:10.3109/13561820902881627 [doi].

40 Fitzpatrick D, Grabarz D, Wang L, et al. How effective is a virtual consultation process in facilitating multidisciplinary decision-making for malignant epidural spinal cord compression?. *Int J Radiat Oncol Biol Phys* 2012;84:e167-72 doi:10.1016/j.ijrobp.2012.03.057 [doi].

41 Tokuda L, Lorenzo L, Theriault A, et al. The utilization of video-conference shared medical appointments in rural diabetes care. *Int J Med Inform* 2016;93:34-41 doi:10.1016/j.ijmedinf.2016.05.007 [doi].

For peer review only

Standards for Reporting Qualitative Research (SRQR)*

http://www.equator-network.org/reporting-guidelines/srqr/

	Answer	Page/line no(s)
**Title and abstract**		
**Title** - Concise description of the nature and topic of the study Identifying the study as qualitative or indicating the approach (e.g., ethnography, grounded theory) or data collection designs (e.g., interview, focus group) is recommended	Does multidisciplinary videoconferencing between a head-and-neck cancer centre and its partner hospital add value to patient care and decision-making? A mixed method evaluation	Title, page 1
**Abstract** - Summary of key elements of the study using the abstract format of the intended publication; typically includes background, purpose, designs, results, and conclusions	The abstract is made conform the layout of BMJ Open: Objectives, Design, Setting, Participants, Primary and secondary outcome measures, Results and Conclusions.	Abstract, page 2
**Introduction**		
**Problem formulation** - Description and significance of the problem/phenomenon studied; review of relevant theory and empirical work; problem statement	Evaluate the video-conferenced MDT as part of the collaboration agreements because it is time consuming and there is the wish to refocus on benefits and drawbacks.	Introduction, page 4-5
**Purpose or research question** - Purpose of the study and specific objectives or questions	Research questions:<br>1. How often are recommendations given on diagnostic and/or therapeutic plans by the teams during video-conferenced MDT?<br>2. What benefits and drawbacks of the videoconference are perceived by the specialists in the teams?	Introduction, page 5
**Designs**		
**Qualitative approach and research paradigm** - Qualitative approach (e.g., ethnography, grounded theory, case study, phenomenology, narrative research) and guiding theory if appropriate; identifying the research paradigm (e.g., postpositivist, constructivist/ interpretivist) is also recommended; rationale**	Thematic, content analysis	Design, page 8-9
**Researcher characteristics and reflexivity** - Researchers' characteristics that may influence the research, including personal attributes, qualifications/experience, relationship with participants, assumptions, and/or presuppositions; potential or actual interaction between researchers' characteristics and the research questions, approach, designs, results, and/or transferability	The researcher is consultant & trainer and is experienced using diverse qualitative analysis and improvement designs. The researcher supports	Design, page 8-9; authors contribution and authors information, page 22

For peer review only - http://bmjopen.bmj.com/site/about/guidelines.xhtml

	as Quality coordinator the University Medical Centre for many care pathways, including the Head & Neck Oncology care pathway.	
**Context** - Setting/site and salient contextual factors; rationale**	The setting is one oncology centre and its partner in the Northern part of the Netherlands. Data of interviews with specialists of the centre and the partner were collected in the clinic.	Abstract, page 2;<br><br>design, page 8
**Sampling strategy** - How and why research participants, documents, or events were selected; criteria for deciding when no further sampling was necessary (e.g., sampling saturation); rationale**	Pre-selected participants were all interviewed: 3 specialists from each team representing all medical specialisms in the videoconferencing. Interviews were performed between 4 and 22 May 2017.	Design, page 8
**Ethical issues pertaining to human subjects** - Documentation of approval by an appropriate ethics review board and participant consent, or explanation for lack thereof; other confidentiality and data security issues	This study was checked by the Medical Ethics Review Board of the UMCG (2016, ref. M16.194909), the Netherlands. They concluded that the study is not a 'clinical research study with human subjects' as meant in the Medical Research Involving Human Subject Act (WMO). Informed consent was not required. The Dutch law requires also a privacy statement from the partner in the study, the Medical Centre Leeuwarden (2016, nWMO 187).	Declarations, page 21
**Data collection designs** - Types of data collected; details of data collection procedures including (as appropriate) start and stop dates of data collection and analysis, iterative process, triangulation of sources/designs, and modification of procedures in response to evolving study findings; rationale**	The semi-structured interviews were audio recorded using a smartphone between May 4 and 22, 2017.	Design, page 8-9; results, page 13.
**Data collection instruments and technologies** - Description of instruments (e.g., interview guides, questionnaires) and devices (e.g., audio recorders) used for data collection; if/how the instrument(s) changed over the course of the study	Interviews were semi-structured using an interview guide; follow up questions were allowed. Participants were also asked to point out improvements.	Design, page 8-9
**Units of study** - Number and relevant characteristics of participants, documents, or events included in the study; level of participation (could be reported in results)	6 specialists participating in the video-conferenced MDT were interviewed.	Design, page 8-9; Results, page 13-17.

For peer review only - http://bmjopen.bmj.com/site/about/guidelines.xhtml

**Data processing** - Designs for processing data prior to and during analysis, including transcription, data entry, data management and security, verification of data integrity, data coding, and anonymization/de-identification of excerpts	Interviews were recorded and transcripts with quotes were returned to the participants for verification. Quotes were anonymized before data-coders started. One data-coder developed codes, a second applied the codes.	Design, page 9
**Data analysis** - Process by which inferences, themes, etc., were identified and developed, including the researchers involved in data analysis; usually references a specific paradigm or approach; rationale**	Major themes were derived from the research questions and minor themes were derived from researcher's field notes during analysis.	Design, page 9
**Techniques to enhance trustworthiness** - Techniques to enhance trustworthiness and credibility of data analysis (e.g., member checking, audit trail, triangulation); rationale**	All results were summarized and were sent to the participants to check credibility using the member check (Synthesized Member Checking, Birt et al. 2016). A 'quality check' of data management was performed by the university hospital Clinical Research Office-department in January 2018.	Design, page 8-9

Results/findings

**Synthesis and interpretation** - Main findings (e.g., interpretations, inferences, and themes); might include development of a theory or model, or integration with prior research or theory	One data-coder developed codes, a second applied the codes. The researcher made the coding tree; that was verified by the coder that developed the codes.	Results, page 13-17
**Links to empirical data** - Evidence (e.g., quotes, field notes, text excerpts, photographs) to substantiate analytic findings	Coding tree in table 4, examples of quotes in results.	Results, page 13-17

Discussion

**Integration with prior work, implications, transferability, and contribution(s) to the field** - Short summary of main findings; explanation of how findings and conclusions connect to, support, elaborate on, or challenge conclusions of earlier scholarship; discussion of scope of application/generalizability; identification of unique contribution(s) to scholarship in a discipline or field	In total 2% recommendations were found in the quantitative part of the study. In interviews with participants of the video-conferenced MDT we found benefits and drawbacks; the specialists recognize that it is important to keep their medical viewpoints aligned and that their patients benefit from the discussions on complex cases; discussing non-complex cases is seen as	Discussion, page 17-21

For peer review only - http://bmjopen.bmj.com/site/about/guidelines.xhtml

	a burden, and the DHCI requirement to discuss all the partner's cases as out-dated.	
**Limitations** - Trustworthiness and limitations of findings	1. The researcher's presence during video conferenced MDT may have influenced the communication between the centre and the partner, also called 'Hawthorne effect' (limitation)<br>2. Only one of the six centres and its preferred partner in the Netherlands was studied (limitation)	Limitations page 2; Discussion, subheading limitations of this study, page 19-20

Other

**Conflicts of interest** - Potential sources of influence or perceived influence on study conduct and conclusions; how these were managed	Competing interests: none declared	page 22
**Funding** - Sources of funding and other support; role of funders in data collection, interpretation, and reporting	None	page 22

*The authors created the SRQR by searching the literature to identify guidelines, reporting standards, and critical appraisal criteria for qualitative research; reviewing the reference lists of retrieved sources; and contacting experts to gain feedback. The SRQR aims to improve the transparency of all aspects of qualitative research by providing clear standards for reporting qualitative research.

**The rationale should briefly discuss the justification for choosing that theory, approach, design, or technique rather than other options available, the assumptions and limitations implicit in those choices, and how those choices influence study conclusions and transferability. As appropriate, the rationale for several items might be discussed together.

Reference:
O'Brien BC, Harris IB, Beckman TJ, Reed DA, Cook DA. **Standards for reporting qualitative research: a synthesis of recommendations.** *Academic Medicine*, Vol. 89, No. 9 / Sept 2014; DOI: 10.1097/ACM.0000000000000388

For peer review only - http://bmjopen.bmj.com/site/about/guidelines.xhtml
